# Comprehensive Detection of Isopeptides between Human Tissue Transglutaminase and Gluten Peptides

**DOI:** 10.3390/nu11102263

**Published:** 2019-09-20

**Authors:** Barbara Lexhaller, Christina Ludwig, Katharina A. Scherf

**Affiliations:** 1 Leibniz-Institute for Food Systems Biology at the Technical University of Munich, Lise-Meitner-Str. 34, 85354 Freising, Germany; b.lexhaller.leibniz-lsb@tum.de; 2 Bavarian Center for Biomolecular Mass Spectrometry (BayBioMS), Technical University of Munich, Gregor-Mendel-Str. 4, 85354 Freising, Germany; tina.ludwig@tum.de; 3 Department of Bioactive and Functional Food Chemistry, Institute of Applied Biosciences, Karlsruhe Institute of Technology (KIT), Adenauerring 20a, 76131 Karlsruhe, Germany

**Keywords:** celiac disease, crosslink, deamidation, gliadin, gluten, isopeptides, LC-MS/MS, tissue transglutaminase, transamidation

## Abstract

Celiac disease (CD) is a chronic inflammation of the small intestine triggered by the ingestion of gluten in genetically predisposed individuals. Tissue transglutaminase (TG2) is a key factor in CD pathogenesis, because it catalyzes both the deamidation of specific glutamine residues and the formation of covalent Nε-(γ-glutamyl)-lysine isopeptide crosslinks resulting in TG2–gluten peptide complexes. These complexes are thought to activate B cells causing the secretion of anti-TG2 autoantibodies that serve as diagnostic markers for CD, although their pathogenic role remains unclear. To gain more insight into the molecular structures of TG2-gluten peptide complexes, we used different proteomics software tools that enable the comprehensive identification of isopeptides. Thus, 34 different isopeptides involving 20 TG2 lysine residues were identified in a model system, only six of which were previously known. Additionally, 36 isopeptides of TG2-TG2 multimers were detected. Experiments with different TG2-gluten peptide molar ratios revealed the most preferred lysine residues involved in isopeptide crosslinking. Expanding the model system to three gluten peptides with more glutamine residues allowed the localization of the preferred glutamine crosslinking sites. These new insights into the structure of TG2-gluten peptide complexes may help clarify the role of extracellular TG2 in CD autoimmunity and in other inflammatory diseases.

## 1. Introduction

Celiac disease (CD) is one of the most frequent food hypersensitivities with a global seroprevalence of 1.4% and a biopsy-confirmed prevalence of 0.7% [1]. This chronic immune-mediated enteropathy of the small intestine is triggered by the ingestion of storage proteins (gluten) from wheat, rye, and barley in genetically predisposed individuals [2]. Due to the high amounts of glutamine (19.7–37.1 mol%) and proline (9.4–23.0 mol%) in the amino acid sequences of gluten proteins [3], the human gastrointestinal enzymes are unable to digest them completely. Thus, peptides with a length of more than nine amino acids reach the small intestinal epithelium [4]. The main genetic factors of CD are the human leukocyte antigen (HLA) class II alleles HLA-DQ2 and HLA-DQ8 of the major histocompatibility complex. Most CD patients (≈90%) carry the HLA-DQ2.5 allele and the remaining patients carry the HLA-DQ8 or HLA-DQ2.2 alleles. These class II molecules are expressed on the surface of B cells and antigen-presenting cells and specifically bind gluten peptides. These peptides are then recognized by CD4+ T cells, which in turn become activated and assist in immunologic processes, like antibody production [4,5].

**Figure 1:**
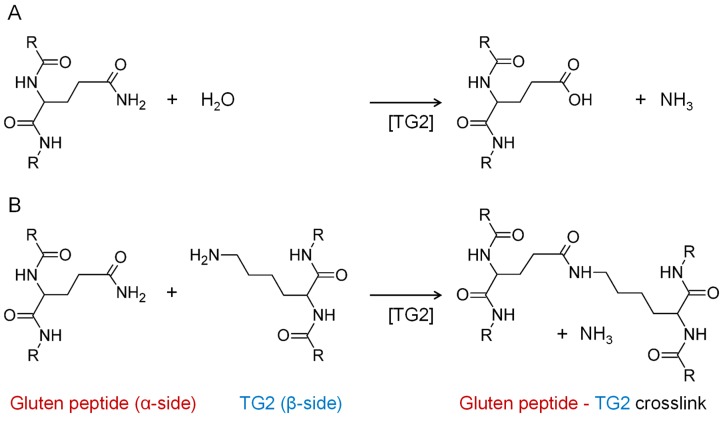
Reactions catalyzed by tissue transglutaminase (TG2). (**A**) Deamidation of glutamine to glutamic acid side chains in the absence of primary amines. (**B**) Crosslinking of glutamine and lysine side chains resulting in the formation of isopeptides.

Human tissue transglutaminase (TG2), a calcium-dependent protein-glutamine γ-glutamyltransferase (EC 2.3.2.13) localized in the cytoplasm is responsible for protein crosslinking, e.g., fibronectin during wound healing [6], and the construction and stabilization of different high-molecular-weight protein structures [7], including crosslinking to collagen [8] and other extracellular matrix components [9]. Extracellular TG2 is implicated in the pathogenesis of a variety of diseases, but due to the complexity of its interactions with other matrix, receptor, cytosolic, and nuclear proteins, the specific contribution of TG2 remains elusive, as does the mechanism by which it is initially secreted from the cell [10]. TG2 catalyzes the deamidation of gluten peptides and converts certain glutamine residues (e.g., QXP or QXXF, where X designates any amino acid) into negatively charged glutamic acid residues, which are a better binding motif for HLA-DQ2.5 leading to enhanced immunogenicity in CD [8]. In addition, TG2 is also responsible for the covalent crosslinking reaction between glutamine and lysine and the resulting formation of Nε-(γ-glutamyl)-lysine isopeptide bonds (Figure 1) [11].

Crosslinking between gluten peptides and TG2 itself as lysine donor is of particular importance, because then TG2-gluten peptide complexes are formed. CD patients’ sera contain anti-TG2 IgA (and IgG or IgM) antibodies [12] and TG2 was identified as the predominant autoantigen of CD [13]. The current models to explain the formation of autoantibodies assume that TG2-specific B cells receive help from gluten-specific CD4+ T cells presented in the context of HLA-DQ2.5 or -DQ8 [14]. Then several routes are possible: (A) according to the original hapten-carrier-model [15], the complexes are taken up by B cell receptors (BCR), the gluten peptide is recognized by gluten-specific CD4+ T cells and these provide help to B cells to secrete anti-TG2 antibodies. (B) Additionally, TG2 may form crosslinks between neighboring BCRs and this could contribute to B cell reactivity. (C) Alternatively, gluten peptides might be crosslinked to the BCRs on the B cell surface by TG2 and thus be directly involved in the uptake and presentation to CD4+ T cells either in the same TG2-BCR complex (D) or with a neighboring BCR [16]. After the BCR-mediated endocytosis of TG2 and the BCR-gluten peptide complexes, TG2 hydrolyzes the isopeptide bond of BCR and gluten peptide and the deamidated peptide is bound immediately to HLA-DQ and presented to CD4+ T cells.

Following the discovery of covalent TG2-gluten peptide complexes, the formation of these complexes was shown in a model system with human TG2 and two model peptides (QLQPFPQPQLPY, PQPQLPYPQPQLPY, binding Q are underlined) derived from α-gliadins. Six lysine residues were shown to be involved in isopeptide bonds with glutamine residues of the model peptides by matrix-assisted laser desorption time-of-flight mass spectrometry (MALDI-TOF MS) and nano-electrospray ionization (ESI)-MS/MS [17]. In addition, TG2 also creates multimers with itself, which can readily incorporate gluten peptides and these complexes might present an antigenic structure in the pathogenesis of CD that eventually triggers autoimmunity [18]. To gain more insights into the molecular structures of TG2-gluten peptide complexes, the identification of isopeptides and especially their crosslinking sites is necessary. Therefore, the overall aim of this work was to identify isopeptides between TG2 and synthetic CD-active gluten peptides in different model systems.

Here we present the identification of 34 isopeptides between human recombinant TG2 and a model peptide derived from α-gliadins and 36 TG2-TG2 isopeptides in TG2 multimers. After identification of the TG2 lysine residues that are involved in crosslinking to the model peptide, the reaction was expanded to three model peptides with more glutamines in their sequences. These synthetic peptides were derived from different gliadin proteins and are known to be immunoreactive in CD with potential crosslinking sites [19,20]. With this extended model system, we were not only able to identify the isopeptides, but it was also possible to obtain information about the localization of the crosslinking sites within the peptides.

## 2. Materials and Methods

### 2.1. Material

All chemicals and solvents were at least HPLC or LC-MS grade. The CD-active model peptide (known immunogenic T-cell epitopes are given in bold [21]) **PFPQPQLPY**-NH_2_ (PepQ; C_54_H_76_N_12_O_12_), derived from α-gliadins, and the model isopeptide standard PFPQPQLPY-NH_2_/NTPSFKER-NH_2_, (with an isopeptide bond at the amino acids Q and K underlined), were purchased from peptides&elephants (Potsdam, Germany) with a purity of >95% and amidated C-termini. The peptides **PQPQLPYPQPQLPY** (P1; C_80_H_116_N_18_O_21_), LQPQQ**PQQSFPQQQ**QPL (P2; C_89_H_138_N_26_O_28_) and VQGQGI**IQPQQPAQL** (P3; C_70_H_117_N_21_O_22_) were obtained from Genscript (Hongkong, PR China) with a purity of >95%. Recombinant human TG2 was purchased from Zedira (Darmstadt, Germany) as a purified and lyophilized protein produced in sf9 insect cells. Trypsin (from bovine pancreas, TPCK-treated, ≥10,000 BAEE U/mg protein) was from Sigma-Aldrich (Steinheim, Germany).

### 2.2. Enzyme Activity Test of TG2

The determination of TG2 enzyme activity was performed with the Tissue Transglutaminase Assay kit (Zedira) [22]. TG2 was diluted 1:10 with deionized water. Analyses of the TG2 sample and the positive control of the test kit were carried out in triplicates against deionized water as blank. The procedure was performed strictly as described by the manufacturer. The absorbances were read at 525 nm with an Infinite M200 microplate reader (Tecan, Salzburg, Austria).

### 2.3. Isopeptide Standard

The isopeptide standard PFPQPQLPY-NH_2_/NTPSFKER-NH_2_ (crosslinked sites are underlined) was dissolved in acetonitrile/water/formic acid (FA) (2:98:0.1) to a concentration of 0.5 ng/µL and directly used for the nLC-MS/MS analysis.

### 2.4. Model Reaction of TG2 and PepQ

The model reaction of TG2 (0.32 nmol/L) with PepQ was performed in Tris-HCl buffer (0.1 mol/L, pH 7.4, 10 mmol/L CaCl_2_) at a molar ratio of TG2:PepQ of 1:150 at 37 °C for 120 min [17]. For inactivation of TG2, all samples were heated at 95 °C for 10 min. The negative controls were prepared by adding PepQ after heat inactivation of TG2. The concentration of 10 mmol/L CaCl_2_ was used, because the information provided by the manufacturer indicated that this concentration is needed to activate human TG2.

### 2.5. Model Reaction at Different Molar Ratios

To study which lysine residues are preferred binding sites, TG2 was incubated with different ratios of PepQ (1:50; 1:10; 1:1; 10:1) in Tris-HCl buffer at 37 °C for 120 min as described above.

### 2.6. Model Reaction with Three Different Model Peptides

The model reaction of TG2 was repeated with the simultaneous addition of the three different peptides P1, P2, and P3. According to the first model reaction, the molar ratios were TG2:P1/P2/P3 of 1:50, respectively. The molar ratios of P1:P2:P3 were 1:1:1. All model reactions were done in triplicates, respectively.

### 2.7. Tryptic Digestion and Clean-Up by Solid Phase Extraction

A trypsin stock solution was added at a trypsin:substrate ratio of 1:100 (*w*/*w*) in 50 mmol/L (NH_4_)_2_CO_3_ to all samples. The solution was incubated at 37 °C for 24 h and the hydrolysis stopped with 3 µL FA to reach a pH value below 2. All samples were purified by solid phase extraction (SPE) using 50 mg Sep-Pak tC18 cc cartridges (Waters, Eschborn, Germany). The C_18_-cartridges were activated with methanol (1 mL), equilibrated with acetonitrile/water/FA (80:20:0.1; 1 mL), and washed with acetonitrile/water/FA (2:98:0.1; 5 × 1 mL). After loading the samples, the cartridges were washed again, and the isopeptides and peptides were eluted with acetonitrile/water/FA (40:60:0.1; 1 mL). The solvent was removed using a vacuum centrifuge (37 °C, 4 h, 800 Pa) and the samples were reconstituted in FA (0.1%, *v*/*v*). Prior to nLC-MS/MS analysis, the peptide concentrations of the reconstituted samples were determined with a NanoDrop Micro-UV–Vis spectrophotometer (NanoDrop One, Thermo Scientific, Madison, WI, USA) at 280 nm. The samples were diluted in the 96-well plates to a concentration of 200 ng/µL with acetonitrile/water/FA (2:98:0.1).

### 2.8. Nanoscale Liquid Chromatography-Tandem Mass Spectrometry

nLC-MS/MS analysis was carried out on an Ultimate 3000 nanoHLPC system (Dionex, Idstein, Germany) coupled to a Q Exactive HF mass spectrometer (Thermo Fisher Scientific, Dreieich, Germany). The nanoscale LC system consisted of a trap column (75 µm × 2 cm, self-packed with Reprosil-Pur C18 ODS-3 5 µm resin, Dr. Maisch, Ammerbuch, Germany) and an analytical column (75 µm × 40 cm, self-packed with Reprosil-Gold, C18, 3 µm resin, Dr. Maisch). After an injection of 5 µL, the peptides were delivered to the trap column using solvent A0 (0.1% FA in water) at a flow rate of 5 µL/min and separated on the analytical column using a 60 min linear gradient from 4% to 32% solvent B at a flow rate of 300 nL/min (solvent A1, 5% DMSO, 0.1% FA in water; solvent B, 5% DMSO, 0.1% FA in acetonitrile) [23]. The MS was operated in data-dependent acquisition mode, automatically switching between MS1 and MS2 spectra. The mass-to-charge (*m/z*) range of the acquisition of the MS1 spectra was 360–1300 *m/z* at an Orbitrap full MS scan (60,000 resolution, 3 × 10^6^ automatic gain control (AGC) target value, 50 ms maximum injection time). In MS2, peptide precursors were selected for fragmentation by higher energy collision-induced dissociation (isolation width of 1.7 Th, maximum injection time of 50 ms, AGC value of 2 × 10^5^). Analysis was performed using 25% normalized collision energy at a resolution of 30,000. For the analysis of the isopeptide standard a maximum injection time of 25 ms, AGC value of 1 × 10^5^ and a resolution of 15,000 was used.

### 2.9. Isopeptide Identification Using MaxQuant

For data analysis, a reciprocal search workflow using one of the most commonly used proteomics software tools MaxQuant (version 1.6.0.1) was developed (Supplemental Figure S1). The Thermo Xcalibur raw files were directly used as input in the MaxQuant software and searched against a human transglutaminase protein database containing 110 entries (UniProtKB, status January 2019) with a peptide-spectrum match (PSM)- and protein-level false discovery rate (FDR) of 1% [24]. All identified tryptic TG2 peptides (Supplemental Figure S2) were filtered for the presence of at least one lysine residue, which resulted in 87 detectable, lysine-containing TG2 peptides. The chemical formulas of these 87 TG2 peptides were calculated (UniProtKB accession no. P21980). Next, we configured these peptides as variable modifications in MaxQuant (TG2-modifications, β-side of the isopeptide, Supplemental Table S1). The used settings were “anywhere” for position, “standard” for type and “Q” for modified amino acid. Theoretical proteases were configured to cleave the model peptides from existing gluten protein sequences with the following cleavage specificities: for PepQ: QP, YP; for P1: FP, YP; for P2: FL, LI; for P3: LV, LE. The parameters were set as follows for the individual search runs: Digestion mode—specific; maximum missed cleavage sites—2; variable modifications—each TG2-modification in one single search run; deamidation at Q; fasta files—UniProtKB accession no. P18573 for PepQ and P1, B6UKP4 for P2, P08453 for P3; contaminant fasta files included; fixed modifications—amidated C-term (only for PepQ); minimum score for modified peptides—10; main search peptide tolerance—4.5 ppm; mass tolerance for fragment ions—20 ppm; all other parameters were used as default settings. To verify the identified isopeptides by reversed search, PepQ (and its deamidated form PepE) were also configured as modifications in MaxQuant (α-side of the isopeptide, PepQ: C_54_H_73_N_11_O_12_, PepE: C_54_H_72_N_10_O_13_) and the raw files were searched against the TG2 sequence with the following parameters: Enzyme—trypsin/P; digestion mode—specific; maximum missed cleavage sites—2; variable modifications—PepQ, PepE; fasta file—UniProtKB accession no. P21980; minimum score for modified peptides—10; main search peptide tolerance—4.5 ppm; mass tolerance for fragment ions—20 ppm; all other parameters were used as default settings. The threshold for unambiguous localization was set to a localization probability of >75%. To confirm the identities of the isopeptides and the identification of the binding site within the isopeptides, the b- and y-fragments of both sides (TG2, β-side and gluten peptides, α-side) were assigned to the respective MS/MS spectra using the software tool MaxQuant Viewer [25].

### 2.10. Assignment of MS/MS Fragments of the Isopeptide Sequences Using ProteinProspector

To further verify the identification of the isopeptides and of the crosslinking sites within the isopeptides, the b-, y- and internal fragments of both sides were calculated with the MS-Product feature of the ProteinProspector webpage (v.5.22.1, University of California, San Francisco, CA, USA) [26]. The sequences of PepQ and the TG2-modifications were entered and the binding Q or K was replaced by “u” for the user-specified amino acid elemental composition of the other isopeptide side, respectively. These “u” compositions for the TG2-modifications were calculated by the formal addition of C_5_H_5_NO_2_ (peptide-bound glutamine minus NH_3_) to the TG2-peptide formulas. For the PepQ modification, the “u” composition was calculated by the formal addition of C_6_H_9_NO (peptide-bound lysine minus NH_3_) to the PepQ formula. ProteinProspector parameters were then set to calculate b-, y- and internal fragments and associated fragments due to water- and ammonia-loss. The charge states were calculated up to 5+ for the precursors and up to 3+ for the fragments.

### 2.11. Isopeptide Confirmation Using Skyline

Skyline (version 4.1.0.11796) was used for confirmation of the identified isopeptides and visualization of label-free peptide precursor chromatograms. PepQ (PFPQ_4_PQ_6_LPY) was modified with an amidated C-terminus at Y and at Q_4_ and Q_6_ either with the TG2-modifications, a deamidation or both to generate the targets, followed by subsequent generation of the appropriate precursors by Skyline. Each PepQ/TG2-modification/deamidation combination was verified according to the following parameters to reject false positively identified isopeptides and confirm confident peak picking: (1) The retention time had to match with the identified retention time of the MaxQuant search, (2) the isotopic dot product score had to be >0.9 (idotp—generated from comparing the expected precursor isotopic distribution to the observed distribution; scored from 0–1, where 1 is the highest) and (3) the comparison of retention time and idotp among the triplicates using the graphical tools had to fit and no detection of the signals in the negative controls had to be observed [27].

### 2.12. Isopeptide Identification Using pLink

For comparative data analysis, the Thermo Xcalibur raw files were directly used as input in the pLink2 software (version 2.3) [28,29] and searched against a user-curated database including the fasta files human tissue transglutaminase (UniProtKB accession no. P21980), the sequence of PepQ for the model system and the fasta files of three gluten proteins (UniProtKB accession no. P18573 for P1, B6UKP4 for P2, P08453 for P3) for the extended model system. The pLink2 search parameters were: Precursor mass tolerance 20 ppm, fragment mass tolerance 20 ppm, cross-linker isopeptide (cross-linking sites K and Q, linker mass −17.031, linker composition N(−1)H(−3)), fixed modification amidated C-term for PepQ, peptide length mininum 6 amino acids and maximum 60 amino acids per chain, peptide mass minimum 600 and maximum 6000 Da per chain, enzyme trypsin, three missed cleavages, FDR ≤ 1% at PSM level.

The raw files of the model system with the three different peptides were analyzed with MaxQuant, Skyline, and pLink2, as described above.

### 2.13. 3D-Structure Model of TG2

To visualize the 3D-structure of TG2 and assign the locations of the crosslinking sites, the sequence models of TG2 in the open conformation (PDB ID code 4PYG) and in the closed conformation (PDB ID code 3S3P) were imported to the 3D graphic software PyMol (The PyMOL Molecular Graphics System, version 2.0 Schrödinger, LLC, New York, NY, USA).

## 3. Results

### 3.1. Determination of TG2 Enzyme Activity

TG2 enzyme activity was analyzed based on the chromogenic hydroxamate detection principle using Z-QQPF as the amine acceptor substrate and hydroxylamine as amine donor [22]. TG2 incorporates hydroxylamine into Z-QQPF to form Z-glutamyl-hydroxamate-QPF that develops a colored complex with iron (III) detectable at 525 nm. The activity of one unit is defined as the amount of enzyme, which causes the formation of 1.0 μmole of Z-glutamyl-hydroxamate-QPF per minute. The activity of TG2 was 2160 units/mg (manufacturer’s certified value: 2554 units/mg). Thus, the TG2 used was confirmed to be active and suitable for all further experiments.

### 3.2. Identification of the Isopeptide Standard

To verify the workflow, the isopeptide standard was measured by nLC-MS/MS and analyzed with MaxQuant. Therefore, PepQ was searched with the NTPSFKER-modification with the same parameters as in the model system. The isopeptide standard was identified with a score of 179.16 and a localization probability of 100% at Q6. MaxQuant identified 21 fragments of the PepQ-side of the isopeptide standard (y_1_; y_2_; y_3_; y_4_; y_5_; y_6_; y_7_; y_8_; y_4_-NH_3_; y_5_-NH_3_; y_7_-NH_3_; y_8_-NH_3_; a_2_; b_2_; b_3_; b_4_; b_5_; b_6_; b_7_; b_8_; b_4_-NH_3_). For the NTPSFKER-side 7 fragments were identified manually (y_2_; y_3_; y_6_; y_7_; b_2_; b_3_; b_5_).

### 3.3. Identification of TG2-Peptides Involved in Isopeptide Formation

In order to identify the lysine residues involved in crosslinking to a glutamine residue of PepQ, MaxQuant searches including variable modifications for all lysine-containing and tryptic TG2 peptides were performed. Altogether, 25 TG2-derived peptides with 20 different lysine residues that are involved in isopeptide formation were identified in the TG2-PepQ model system. To verify the identified isopeptides, the data was also searched with the crosslinking software tool pLink2. Table 1 shows the identified isopeptides with the lysine positions in the amino acid sequence of TG2, the sequences of the identified TG2 peptides (β-side of the isopeptide) with the crosslinking lysine residues, the sequence of PepQ and the deamidated form of PepQ (called PepE in the following, α-side of the isopeptide) with the crosslinking glutamine residues and additional deamidation sites, as well as the *m/z* values of the precursor ions and their charge states and the pLink2 identification E-value.

The crosslinking sites in PepQ/PepE were localized unambiguously in almost all identified isopeptides (28) (Table 1A) except for two isopeptides (K-464, K-550), where the exact localization of the crosslinking site was unclear in the gluten peptide (Table 1B). In four isopeptides, the crosslinking sites were localized unambiguously for PepQ/PepE, but remained unclear in the TG2 peptides (K-598/600, K-600/602, 2 × K-672/674).

**Figure 2:**
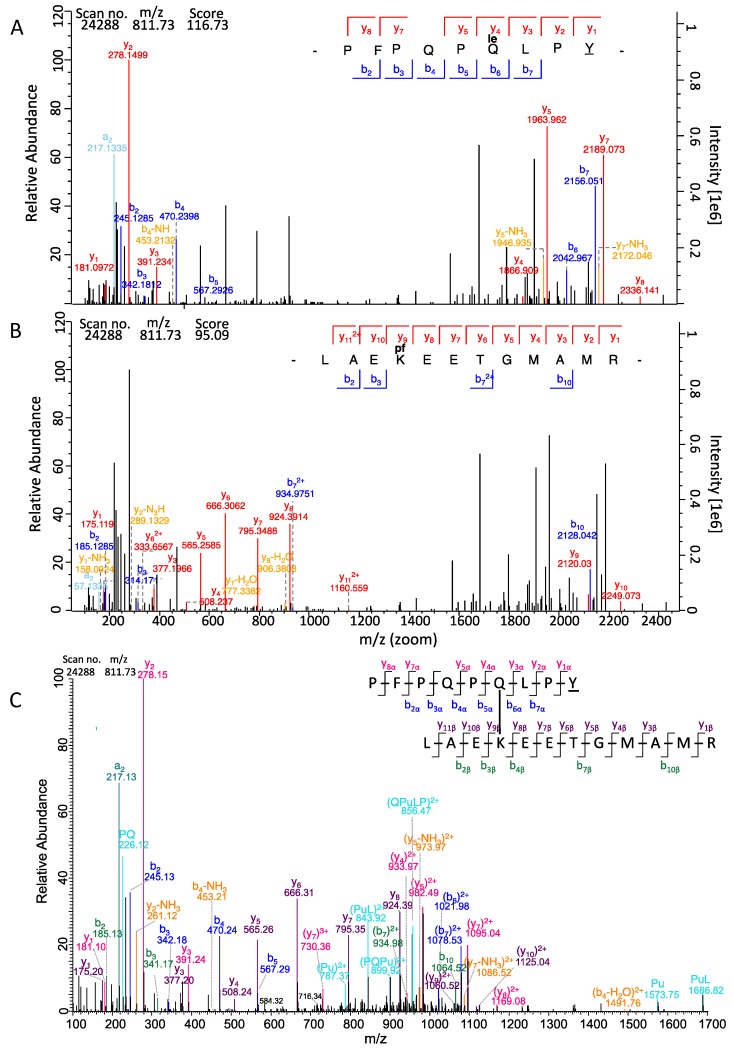
MS/MS spectrum (scan no. 24288) of the isopeptide between LAEKEETGMAMR (TG2) and PFPQPQLPY (PepQ). (**A**) Spectrum of the isopeptide with fragments of PepQ carrying the TG2 peptide as modification annotated by MaxQuant Viewer. (**B**) Spectrum of the isopeptide with fragments of LAEKEETGMAMR carrying PepQ as modification annotated by MaxQuant Viewer. The fragments are marked as follows: y-fragments in red; b-fragments in blue; a- and c-fragments in turquoise; fragments with losses of NH_3_ or CO marked in orange. (**C**) Spectrum of the isopeptide annotated manually with fragments of both sides of the isopeptides, calculated with Protein Prospector. The fragments are marked as follows: y-fragments of PepQ in red; b-fragments of PepQ in blue; a- and internal fragments in turquoise; y-fragments of the TG2 peptide in violet; b-fragments of TG2 peptides in green (single amino acids); fragments with losses of NH_3_ or CO marked in orange.

Since two reciprocal data analysis steps were performed, two scores were received for each isopeptide that differed from one another in most cases. The α-score was calculated from the search against the α-gliadin fasta for PepQ carrying either TG2-modification, whereas the β-score was calculated from the reversed search against the TG2 fasta for TG2 peptides carrying PepQ or PepE as modification (Table 1). One of the crosslinked peptides in the isopeptide (in most cases PepQ/PepE) often fragmented better than the other one thus resulting in different scores [26]. We defined on the basis of the data that one of the two isopeptide scores should be >100 and the other one >40 (default setting for modified peptides in MaxQuant) as a threshold for confident identification. The isopeptides DLYLENPEIKIR/PepQ with the scores 164.89 (α-side) and 183.03 (β-side) and EDITHTYKYPEGSSEER/PepQ with the scores 133.60 (α-side) and 141.60 (β-side) were the highest scoring isopeptides in our analysis. Further, we manually curated the MS/MS spectra of all isopeptides using the MaxQuant Viewer. For that, the highest scoring MS/MS scan number per identified isopeptide was loaded into the Viewer tool. The signals were annotated with the b- and y-fragments. The isopeptide PepQ/LAEKEETGMAMR is highlighted as an example in Figure 2. First, the MaxQuant search result of PepQ carrying the TG2 isopeptide modification “**le**” (=LAEKEETGMAMR) at Q6 was loaded and all annotated y- and b-ion fragments were highlighted (Figure 2A). In Figure 2B the identified reverse isopeptide was loaded into the MaxQuant Viewer, so here the b- and y-fragments of the LAEKEETGMAMR peptide carrying the PepQ modification at K is shown (Figure 2B). For confirmation, a further annotation was done manually by combining the information from both spectral annotations (Figure 2C). Here not only the fragment ions annotated by MaxQuant (msms.txt output file) [24] were indicated, but also internal fragment ions (double fragmentation on both crosslinked peptide sequences) calculated with the MS-Product feature of ProteinProspector [26].

### 3.4. Identification of TG2-TG2 Crosslinks

Previous studies showed that multiple glutamine and lysine residues of TG2 were involved in TG2-TG2 self-crosslinking [18]. Using the pLink2 software for the verification of the TG2-PepQ isopeptides, the TG2 multimers were identified parallel with an E-value <0.01 and at least detected in two or more MS^2^ scans. In the model system 36 different TG2-TG2 isopeptides were identified (Table 2), whereas 8 combinations of the crosslinked sites were known [18].

### 3.5. Identification of Crosslinks and Deamidation Sites Within PepQ

TG2 performs both crosslinking and deamidation reactions at glutamine residues. PepQ has two glutamine residues in its sequence, which can be either crosslinked or deamidated. Vader et al. [30] showed that the Q is no target for TG2 in a QP sequence, but that the sequence QXP is a very good target for TG2 due to the neighboring C-terminal amino acids. The model peptide with only deamidation on one or two Qs and no crosslinking modification were also identified with the default MaxQuant search settings. The localization probabilities for the crosslinking and deamidation were obtained with MaxQuant and verified with pLink2. The isopeptide crosslinking site in PepQ was located at Q_6_ in all identified isopeptides with probabilities for the correct identification ranging from 97.2–100% (Table 1). To verify the identified isopeptides, the data were additionally searched with pLink2 and the resulting E-values are also given in Table 1. Crosslinking sites were unambiguously identified for almost all isopeptides with PepQ (Supplemental Figure S3). One example is shown in Figure 2A for the isopeptide PepQ/LAEKEETGMAMR, for which the specific fragments b_5_, b_6_, y_5_, and y_4_ as the relevant fragment ions for unambiguous site determination were all confidently detected.

Considering the deamidated form PepE, the localization of the crosslinking site varies between Q_4_ and Q_6_, but the probabilities for correct site localization were 95.8–100%. For example, in the isopeptide FLKNAGR/PepE, the isopeptide probability was 99.7% for Q_6_, and the deamidation probability for Q_4_ was 99.7% as well, because there are only two Q’s present in the peptide sequence. These findings indicate that in the isopeptides with a deamidated Q_6_ (7 isopeptides detected, see Table 1), TG2 most likely first deamidated the preferred Q and subsequently built the isopeptide bond with Q_4_. In the isopeptides with a deamidated Q_4_ (4 isopeptides detected, see Table 1), probably the isopeptide bond was first formed by TG2 on Q_6_, and the additional deamidation may have been caused by a non-enzymatic process due to the basic pH conditions of the tryptic digestion protocol [31]. Spontaneous deamidation of PepQ at either Q_4_ or Q_6_ and less frequently at both sites was detected in the control experiments with inactivated TG2, indicating that the non-enzymatic process did occur under the reaction conditions used in this study.

Table 1B shows the two isopeptides with ambiguous crosslinking sites of PepQ and PepE. ANHLNKLEAK/PepE has a localization probability of 50% for Q_4_ or Q_6_ within PepE, because all specific fragments (b_5_, b_6_, y_5_, and y_4_) were absent (Supplemental Figure S3R). For SVPLCILYEKYR/PepQ no determination of localization probabilities was possible, because this isopeptide was only identified from its β-side (Supplemental Figure S3S).

### 3.6. Visualization of Isopeptides with Skyline

In order to confirm and visualize the MS1 precursor chromatograms of all isopeptides, we used the software tool Skyline [32] to confirm both sides of the isopeptides. TG2-lysine peptides carrying crosslinked PepQ/PepE as well as PepQ/PepE carrying crosslinked TG2-lysine peptides were likewise investigated with Skyline. Each isopeptide was confirmed by retention time, idotp value, and the comparison between samples and negative controls, i.e., we obtained a Gaussian peak shape in the samples for all isopeptides, but no signal (intensity <1 × 10^2^) in the negative controls.

### 3.7. Estimation of Preferred Lysine Residues

**Figure 3:**
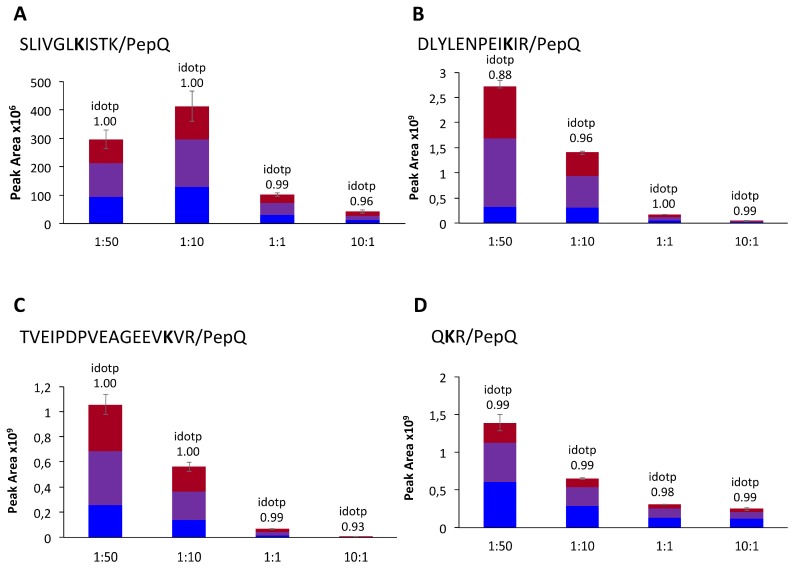
Peak areas of the isopeptides between PepQ and the most preferred TG2 binding sites. The illustration shows the peak areas for the isopeptides (**A**) SLIVGLKISTK/PepQ, (**B**) DLYLENPEIKIR/PepQ, (**C**) TVEIPDPVEAGEEVKVR/PepQ and (**D**) QKR/PepQ in the molar ratios 1:50, 1:10, 1:1, and 10:1, respectively.

To study the preferred binding sites within the TG2-PepQ complexes, the model system was expanded to a total of five different TG2:PepQ molar ratios (1:150; 1:50; 1:10; 1:1; 10:1). By comparing the intensities of the signals of the five ratios for every isopeptide individually, the most preferred lysine residues were identified. For the isopeptides SLIVGLKISTK/PepQ, QKR/PepQ, TVEIPDPVEAGEEVKVR/PepQ, and DLYLENPEIKIR/PepQ, a signal with a peak area >1 × 10^6^ and a Gaussian peak shape was already monitored at the ratio 10:1 and with increasing TG2-PepQ ratios the peak areas also increased (Figure 3).

Thus, the four lysine residues K-425, K-590, K-600, and K-649 were the most preferred crosslinking sites in the model reaction. For the less preferred lysine residues (K-205, K-464, K-562, K-598, K-672, K-677) within the identified isopeptides, signals were observed first in the 1:1 or 1:10 ratio samples with a peak area >1 × 10^6^ (data not shown). Isopeptides with least preferred lysine residues only showed signals in the model system with the highest amount of PepQ (1:150) (data not shown, for an overview of crosslinking sites in the TG2 sequence, see Supplemental Figure S4).

### 3.8. Location of the Complex-Forming Lysine Residues in the 3D-Model of TG2

**Figure 4:**
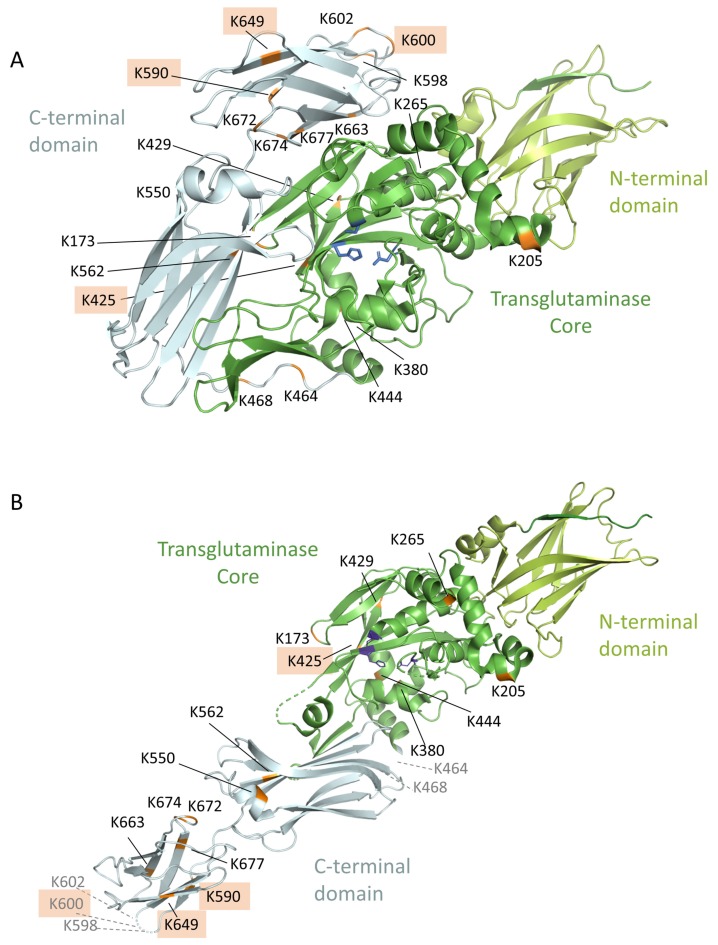
Location of the lysine residues identified crosslinked with the model peptide PepQ within the 3D structure of TG2. (**A**) 3D structure of the closed conformation (PDB ID code 4PYG). (**B**) 3D structure of the active open conformation (PDB ID code 3S3P); the lysine residues K464, K468, K598, K600, and K602 are not visible in this scheme, because these parts are not resolved in the crystal structure. The identified lysine residues are marked in orange, the four preferred lysine residues are highlighted, the catalytic triad is colored in blue, the C-terminal domain in grey, the core region in dark green, and the N-terminal region in light green.

As previously demonstrated by Stamnaes et al. [18], the 3D-structure of TG2 shows an accumulation of the binding lysine residues in the C-terminal domain and in the catalytic core with the active site C-277, H-335 and D-358 (Figure 4). Most of these lysine residues are located in close proximity to the active site or at exposed sites, especially in the active open conformation. The N-terminal domain of the enzyme seems to remain without modified lysine residues in the most cases, which is important, because the epitopes that are recognized by the anti-TG2 antibodies are located in this region and are not blocked [33].

### 3.9. Identification of Isopeptides in the Extended Model System

The model system was extended to three different gluten peptides and the samples were analyzed with the described workflow with MaxQuant and Skyline as well as with pLink2. The deamidated forms of the model peptides were also identified without crosslinking modifications. The observed isopeptides within the extended model system were used to identify the preferred glutamine crosslinking sites in the gluten peptides. Table 3, Table 4 and Table 5 show all identified isopeptides with their MaxQuant scores and crosslinking probabilities for every glutamine within the gluten peptide sequence as well as with the pLink2 E-values and the number of MS^2^ scans they are identified with. Parts A of the tables report the isopeptides with unambiguous localization of the crosslinking and deamidation sites, Parts B show the ambiguous ones, and Parts C the isopeptides that were only identified with pLink2.

For P1, the most preferred glutamine was Q_11_ for the isopeptide bond, and Q_4_ for the deamidation. For the exceptional cases when the crosslinking site was located at Q_4_ (3×) always one deamidation was situated at Q_11_. When the crosslinking site was located at the unusual target Q_9_P, the most preferred deamidated sites were Q_11_ and Q_4_. For identifications with a higher score, the probabilities for one specific position are also higher. These probabilities depend on whether the specific fragment ions around the crosslinking sites were identified or not. In peptide sequences with glutamine residues located close together, the fragments between them were often not identified. Some isopeptides were identified only by the workflow with MaxQuant (6×). Others were only found with pLink2 (4×) from MS^2^ scans with a low intensity. The isopeptide bond in P2 tended to be at Q_2_, Q_10_ or Q_14_ and the deamidated glutamine residues were mostly at Q_4_, Q_10_ and Q_14_. Q_10_ represents the motif Q_10_XP, which is known as a preferred glutamine residue for a modification by TG2. In all isopeptides involving P2 this preferred Q_10_ was either crosslinked (10×) or at least deamidated (21×). The unusual TG2 target Q_8_P was involved in isopeptide formation in just a few cases (2×). The localization probabilities for the crosslinking and deamidation sites for the isopeptides with P2 were almost all unambiguously identified. The position of the isopeptide bond within P3 was observed more in the front part of the peptide at Q_4_ (10×) or Q_7_ (9×), whereas Q_4_ is known as a good target for TG2 [30]. For these isopeptides in which the crosslinking site was located in the rear part, it was always at Q_12_ (7×), which is also known as a good TG2 motif. In turn, the deamidation took place more in the rear part of the sequence at Q_12_, Q_13_, or Q_14_, whereby Q_12_ and Q_14_ are known as motif with an increasing effect on TG2 modification activity, but Q_13_ with an decreasing effect [30].

**Figure 5:**
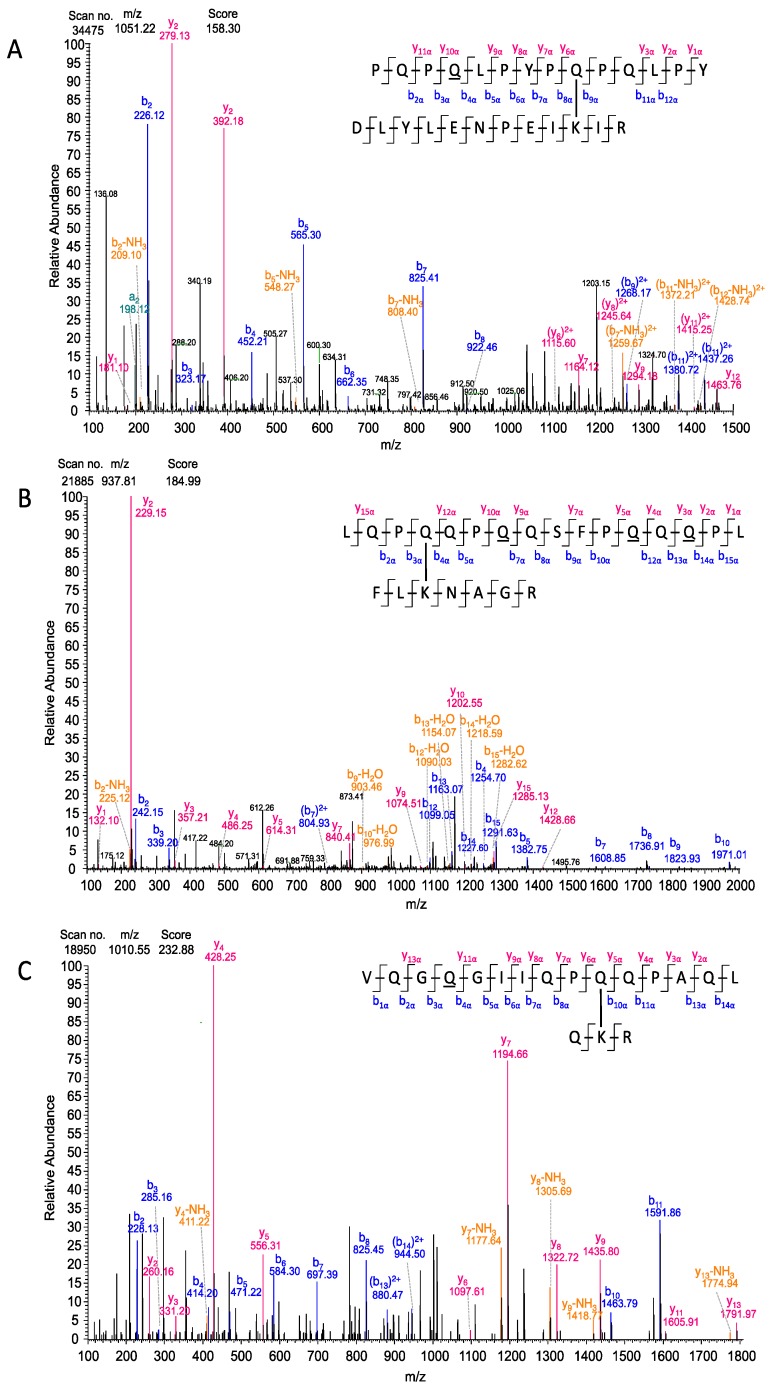
MS/MS spectra of the isopeptides between the three model peptides of the advanced model system and TG2. (**A**) Spectrum of the isopeptide between peptide P1 PQPQLPYPQPQLPY crosslinked to TG2 peptide DLYLENPEIKIR and annotated with fragments of P1 by MaxQuant Viewer. (**B**) Spectrum of the isopeptide between peptide P2 LQPQQPQQSFPQQQPL and TG2 peptide FLKNAGR annotated with fragments of P2 by MaxQuant Viewer. (**C**) Spectrum of the isopeptide between peptide P3 VQGQGIIQPQQPAQL crosslinked to TG2 peptide QKR annotated with fragments of P3 by MaxQuant Viewer. The fragments are marked in different colors as follows: y-fragments in red; b-fragments in blue; fragments with losses of NH_3_ or CO marked in orange. The deamidated glutamine residues are underlined.

These results were further confirmed by annotation of MS/MS spectra with the series of b- and y-fragments from the MaxQuant output tables (msms.txt files). One isopeptide each was chosen as an example for each model peptide (Figure 5). Fragmentation within the model peptides resulted in almost the whole b- and y-series. In the MS/MS spectrum of P1/DLYLENPEIKIR involving K-590 (Figure 5A) the most intense signals were annotated with the smaller b- and y-fragments. The MS/MS spectrum for P2/FLKNAGR involving K-205 (Figure 5B) showed most signals in the higher *m/z* range and they were annotated with the b-fragments. The fragments in the MS/MS spectrum for P3/QKR involving K-600 (Figure 5C) were distributed over the entire *m/z* range with equally intensive signals for the b- and y-series. Due to the identification of specific fragments around the crosslinking site, the crosslinking glutamine residues were unambiguously confirmed for this isopeptide.

## 4. Discussion

In this study we used a workflow with the proteomics tool MaxQuant, its integrated search engine Andromeda and Skyline as well as the crosslinking software tool pLink2 to identify isopeptides between TG2 and gluten-derived model peptides. When using these tools, the whole computational part is run without a client-server on the user’s computer [34]. We have demonstrated a workflow to identify enzymatically built isopeptides as well as the localization of the crosslinking site within these peptides. In some cases, an unambiguous identification was not possible, because of missing specific fragment ion information, but the localization probability could still be limited to a short part of the peptide sequence. In total, we identified 34 isopeptides with 20 different lysine residues as crosslinking sites. Six of these crosslinking sites were already known as TG2-gluten peptide binding sites [17] and eleven as lysine residues involved in TG2 multimer self-crosslinking [18]. In the model system, 36 TG2-TG2 isopeptides were additionally detected with their crosslinking glutamine and lysine residues. Nine of these TG2-TG2 isopeptide crosslinking combinations were already known [18], as well as six of the nine identified glutamine and nine of the eleven identified lysine residues involved in TG2-multimerization. Furthermore, the four most preferred binding lysine residues (K-425, K-590, K-600 and K-649) in the model system were identified by analyzing different TG2:PepQ ratios. K-590, K-600, K649 were already known as crosslinking sites [17] and are located in the C-terminal domain of TG2. K-425 was shown to be involved in TG2 self-multimerization [18] and is part of the core region next to the catalytic core. All four lysine residues are exposed positions according to the published structures in the PDB database (Figure 4).

TG2 performs both crosslinking and deamidation of glutamine residues. The model peptide PepQ (PFPQ_4_PQ_6_LPY) comprises two possible targets, Q_4_ and Q_6_, for TG2. We identified isopeptides without deamidation of the second glutamine in PepQ and some with deamidated PepQ (Table 1) at either Q_4_ or Q_6_. Vader et al. [30] investigated the TG2 deamidation pattern depending on the neighboring C-terminal amino acid. Applied to PepQ, the motif PQ_6_L is a good target and the motif PQ_4_P a weak target for deamidation by TG2. The crosslink formation took place at the expected target Q_6_ in the isopeptides without deamidation. In earlier studies of Dorum et al. [21], Q_6_ was identified as a crosslinking target for TG2. When looking at the PepE sequences within isopeptides, the crosslink was almost always at Q_4_ and the deamidation at Q_6_. In this case and in keeping with previous findings [21,30], these data may indicate first deamidation at the preferred Q_6_ followed by crosslinking at the less preferable Q_4_, both reactions implemented by TG2. In case of deamidated Q_4_, the results indicate crosslinking by TG2 followed by a non-enzymatic deamidation due to the alkaline pH conditions during tryptic digestion [31]. The deamidation of the glutamine residues in only deamidated model peptides may be caused by TG2 and additionally the pH conditions. One limitation of the current experimental design is that it does not allow a clear differentiation between enzymatic and non-enzymatic deamidation, because the original intent was to focus on the identification of crosslinking sites, rather than deamidation sites. Further experiments would be necessary to look more closely into the specific mechanisms of crosslinking versus deamidation. The localization probabilities of the modifications are given due to the measurement of specific fragments situated around the targets. This leads to probabilities <75% in some cases, when some of these fragments are missing. In these cases, only the subpart of the sequence can be identified where the possibly crosslinked glutamine residues are located.

It is well established that TG2 is very specific in its deamidation pattern [30], which can be explained by strong effects of the neighboring C-terminal amino acids. In our expanded model system with three gluten model peptides, we demonstrated that TG2 follows the known selective deamidation pattern in almost all deamidated isopeptides. Only in a few cases, where the identification scores were low or the specific fragments were absent, it was not feasible to determine the unambiguous localization of the deamidation. The crosslinking reaction also depends on this selectivity of TG2, but with more exceptions. For the shorter model peptide P1 with less glutamine residues, most of the crosslinking sites within the isopeptides were identified clearly due to the presence of the specific fragments. For the longest peptide P2 with nine glutamine residues, the identification of one specific crosslinking site was more difficult, especially when the isopeptides were identified with a low score. In these cases, it was nevertheless possible to identify the subpart within the peptide sequence that most likely carries the modification. These findings underscore partly the known TG2 selectivity by showing a clear preference for the deamidation pattern. Our data also demonstrate a difference in the crosslinking selectivity or at least a dependence on the previously deamidated glutamine residues.

The alignment of crosslinking sites of TG2 (Supplemental Table S2) including the four surrounding amino acids in both C- and N-terminal direction did not reveal an obvious pattern regarding preferred chemical environments around the reactive sites in the primary structure. Therefore, it seems likely that the secondary structure of TG2 is more important to determine which lysine residues are preferred crosslinking sites. Further experiments, e.g., using amino acid substitution analysis on recombinant TG2 in combination with computational modelling, would be useful to get more detailed insights into secondary structural elements that predict which lysine residues are reactive crosslinking sites and which ones are not.

In summary, using a reciprocal search workflow with commonly used proteomics tools and a recently developed crosslinking tool helps to identify many isopeptides with a high certainty and a few isopeptides just with one of the strategies. These novel insights into the molecular structures of TG2-gluten peptide complexes may help clarify the function of extracellular TG2 in the initiation of CD autoimmunity and the role of anti-TG2 autoantibodies. To shed more light on the immunological and physiological relevance of these complexes, in vivo experiments on the extent and the activation of B cells are necessary. Further experiments together with partners bringing in complementary expertise, especially in immunology, would be needed to address the most relevant point regarding the link between our findings and TG2-mediated gluten peptide presentation in CD. Crosslinking reactions are implicated in a number of inflammatory diseases, degenerative disorders, and even cancer, so this strategy may open up multiple opportunities for further research. Future efforts will aim to determine isopeptides of TG2 with physiologically relevant gluten hydrolysates from wheat, rye, and barley.

## Figures and Tables

**Table 1:** Lysine residues of TG2 involved in the formation of 34 different isopeptides with the model peptide PepQ (PFPQ_4_PQ_6_LPY).

**(A) TG2-PepQ Isopeptides with Unambiguous Modification Sites (Localization Probability >75% for the Binding Site in PepQ)**										
**Lysine Residue ^a^**	**Sequence of TG2 peptide**	**Modification**	**Precursor**	**Charge State**	**MaxQuant Score**		**No. of Fragment Matches**		**Q6 Isopeptide Probabilities**	**Identification E-value pLink2 ^b^**
	**β-side ^c^**	**α-side ^d,e^**	***m*/*z***		**α ^f^**	**β ^g^**	**α**	**β**	**[%]**	
**173**	QEYVLTQQGFIYQGSAKFIK	PFPQPQLPY	1139.59	3+	30.17	128.14	6	32	4.2	4.40 × 10^−4^
**205**	FLKNAGR	PFPQPQLPY	625.01	3+	135.77	35.63	18	10	100	2.24 × 10^−11^
		PFPEPQLPY	625.34	3+	117.16	19.92	15	8	99.7	-
**265**	WKNHGCQR	PFPQPQLPY	699.36	3+	79.45	29.81 ^h^	12	4	100	6.36 × 10^−6^
**380**	AIKEGDLSTK	PFPQPQLPY	1065.07	2+	151.17	29.74	17	7	100	6.56 × 10^−7^
		PFPQPELPY	710.71	3+	94.14	45.88	14	14	1.7	-
**425**	SLIVGLKISTK	PFPQPQLPY	742.77	3+	79.94	84.48	12	21	98.0	7.57 × 10^−6^
**429**	ISTKSVGR	PFPQPQLPY	639.02	3+	107.90	26.96 ^h^	17	5	100	4.57 × 10^−6^
**429**	ISTKSVGRDER	PFPQPQLPY	579.56	4+	168.23	- ^i^	22	- ^h^	100	-
**444**	EDITHTYKYPEGSSEER ^j^	PFPQPQLPY	1036.82	3+	133.60	141.60	17	33	100	2.74 × 10^−8^
		PFPQPELPY	1037.15	3+	119.29	96.45	14	29	0.3	-
**444**	DEREDITHTYKYPEGSSEER	PFPQPQLPY	877.91	4+	63.57	53.59	9	17	89.7	-
**464**	ANHLNKLAEK	PFPQPQLPY	735.73	3+	155.82	47.98	22	16	100	-
**464**	ANHLNKLAEKEETGMAMR	PFPQPQLPY	622.92	5+	110.31	92.31	17	25	100	1.07 × 10^−6^
		PFPEPQLPY	623.11	5+	121.99	78.02	14	22	0.2	-
**468**	LAEKEETGMAMR	PFPQPQLPY	811.73	3+	116.73	95.09	17	21	100	6.50 × 10^−11^
		PFPEPQLPY	812.06	3+	73.93	38.77	12	12	99.5	-
**562**	DCLTESNLIKVR ^j^	PFPQPQLPY	820.10	3+	92.47	199.82	14	31	100	3.01 × 10^−8^
**562**	YRDCLTESNLIKVR	PFPQPQLPY	695.12	4+	157.86	111.74	19	26	100	-
**590**	DLYLENPEIKIR ^j^	PFPQPQLPY	1285.67	2+	164.89	183.03	19	28	100	2.62 × 10^−6^
		PFPQPELPY	1286.18	2+	59.21	108.56	8	21	13	-
**598**	ILGEPKQK	PFPQPQLPY	660.73	3+	118.01	57.18 ^h^	14	10	99.9	5.38 × 10^−7^
**598**	ILGEPKQKR	PFPQPQLPY	712.74	3+	108.64	51.95	15	12	100	3.56 × 10^−7^
**600**	QKR ^k^	PFPQPQLPY	749.91	2+	195.98	- ^i^	20	-^h^	100	-
**649**	TVEIPDPVEAGEEVKVR ^i^	PFPQPQLPY	978.85	3+	29.16	122.10	10	24	-	1.04 × 10^−2^
**663**	MDLLPLHMGLHKLVVNFESDKLK	PFPQPQLPY	937.01	4+	93.20	103.02	15	32	98.5	-
**677**	AVKGFR ^j^	PFPQPQLPY	872.98	2+	168.74	22.29 ^g^	19	7	100	1.04 × 10^−6^
		PFPQPELPY	873.47	2+	151.51	- ^i^	17	-^h^	0	-
**(B) TG2-PepQ Isopeptides with Ambiguous Modification Sites**										
**464**	ANHLNKLAEK	PFPEPQLPY	552.30	4	53.17	48.28	9	14	50	-
**550**	SVPLCILYEKYR	PFPQPQLPY	851.12	3	- ^i^	47.92	- ^i^	11	-	-
**598/600**	ILGEPKQK ^l^	PFPQPELPY	661.03	3+	98.30	65.25	12	10	6.8	-
**600/602**	QKRK^j k,l^	PFPQPQLPY	542.98	3+	121.99	- ^i^	15	- ^h^	100	-
**672/674**	LVVNFESDKLK	PFPQPQLPY	787.09	3+	104.75	103.91	17	20	100	8.51 × 10^−7^
**672/674**	LVVNFESDKLKAVK ^h,l^	PFPQPQLPY	665.12	4+	50.70	52.27	9	13	97.2	1.57 × 10^−2 b^

^a^ Position of the lysine residue in the amino acid sequence of human tissue transglutaminase (TG2, P21980); ^b^ pLink2 E-value <0.01 [28]; ^c^ Lysine residue involved in isopeptide formation is underlined (isopeptide localization probability >95%); ^d^ Glutamine residue involved in isopeptide formation is underlined; ^e^ Formation of glutamic acid through deamidation activity of TG2; ^f^ MaxQuant score calculated from the search against the α-gliadin fasta (P18573) for PepQ carrying either TG2-modification; ^g^ MaxQuant score calculated from the reversed search against the TG2 fasta (P21980) for TG2 peptides carrying PepQ or PepE as modification; ^h^ Isopeptide side were identified with different *m*/*z* values; ^i^ Not detected; ^j^ Isopeptide already identified previously by Fleckenstein et al. (2004) [17]; ^k^ β-Sequence too short to be identified by MaxQuant; ^l^ The exact position of the binding lysine residue was not detectable due to missing fragments; the Q_4_ deamidation probability is calculated by 100% minus the Q_6_ deamidation probability.

**Table 2:** Lysine residues and glutamine residues of TG2 involved in the formation of TG2 multimers.

Sequence of Isopeptide ^a^	Precursor	Charge State	Residues in TG2 ^b^		E-value pLink2 ^c^	Number of MS^2^ Scans ^d^
	*m*/*z*		K	Q		
**VVSGMVNCNDDQGVLLGR/EKLVVR**	2601.35	4+	30	234	2.46 × 10^−12^	6
**VVSGMVNCNDDQGVLLGR/FLKNAGR**	2663.34	3+	205	234	1.77 × 10^−17^	10
**QEYVLTQQGFIYQGSAK/AIKEGDLSTK**	3003.53	3+	380	164	3.48 × 10^−15^	3
**VVSGMVNCNDDQGVLLGR/AIKEGDLSTK**	2919.45	3+	380	234	1.96 × 10^−15^	5
**AIKEGDLSTK/NHGCQR**	1757.86	4+	380	270	1.50 × 10^−4^	6
**NEFGEIQGDK/AIKEGDLSTK**	2180.07	3+	380	324	7.94 × 10^−7^	2
**VVTNYNSAHDQNSNLLIEYFR/AIKEGDLSTK ^e^**	3540.76	4+	380	307	2.23 × 10^−7^	6
**VVSGMVNCNDDQGVLLGR/ISTKSVGRDER**	3105.54	5+	429	234	1.07 × 10^−3^	3
**EDITHTYKYPEGSSEER/VVSGMVNCNDDQGVLLGR**	3898.79	5+	444	234	1.62 × 10^−8^	6
**EDITHTYKYPEGSSEER/WKNHGCQR**	3051.36	5+	444	270	4.24 × 10^−3^	2
**VVTNYNSAHDQNSNLLIEYFR/EDITHTYKYPEGSSEER**	4520.09	6+	444	307	3.36 × 10^−12^	7
**EDITHTYKYPEGSSEER/NEFGEIQGDK**	3159.40	5+	444	324	1.94 × 10^−3^	4
**VGQSMNMGSDFDVFAHITNNTAEEYVCR/EDITHTYKYPEGSSEER ^e^**	5158.24	5+	444	481	3.54 × 10^−11^	3
**VVSGMVNCNDDQGVLLGR/ANHLNKLAEK**	2995.51	4+	464	234	2.93 × 10^−9^	4
**VVTNYNSAHDQNSNLLIEYFR/ANHLNKLAEK ^e^**	3616.81	5+	464	307	1.20 × 10^−6^	10
**VGQSMNMGSDFDVFAHITNNTAEEYVCR/ANHLNKLAEK**	4254.96	5+	464	481	9.92 × 10^−8^	5
**VGQSMNMGSDFDVFAHITNNTAEEYVCR/LEAKEETGMAMR ^e^**	4482.98	5+	468	481	1.20 × 10^−17^	4
**QEYVLTQQGFIYQGSAK/DLYLENPEIKIR**	3444.77	4+	590	164	6.77 × 10^−5^	3
**VVSGMVNCNDDQGVLLGR/DLYLENPEIKIR**	3360.69	4+	590	234	7.07 × 10^−11^	7
**DLYLENPEIKIR/WKNHGCQR**	2513.27	4+	590	270	2.01 × 10^−11^	5
**VVTNYNSAHDQNSNLLIEYFR/DLYLENPEIKIR**	3981.99	5+	590	307	3.04 × 10^−7^	4
**ILGEPKQK/AVKGFR**	1571.92	4+	599	677	1.03 × 10^−10^	4
**ILGEPKQKR/AVKGFR**	1728.03	4+	599	677	1.21 × 10^−3^	3
**VVSGMVNCNDDQGVLLGR/TVEIPDPVEAGEEVKVR ^e^**	3724.85	4+	649	234	6.78 × 10^−13^	6
**TVEIPDPVEAGEEVKVR/NHGCQR**	2563.26	4+	649	270	5.69 × 10^−3^	2
**VVTNYNSAHDQNSNLLIEYFR/TVEIPDPVEAGEEVKVR**	4346.16	5+	649	307	7.66 × 10^−5^	4
**LVVNFESDKLKAVK/NHGCQR**	2286.20	5+	672	270	8.80 × 10^−6^	2
**QEYVLTQQGFIYQGSAK/AVKGFR**	2619.36	4+	677	164	5.82 × 10^−9^	6
**QEYVLTQQGFIYQGSAK/FLKNAGR**	2747.42	3+	677	164	8.31 × 10^−11^	4
**QEYVLTQQGFIYQGSAK/TVEIPDPVEAGEEVKVR**	3808.93	6+	677	164	8.33 × 10^−6^	2
**QEYVLTQQGFIYQGSAK/AVKGFR**	2619.36	4+	677	169	5.15 × 10^−7^	10
**VVSGMVNCNDDQGVLLGR/AVKGFR ^e^**	2535.28	4+	677	234	1.70 × 10^−11^	14
**WKNHGCQR/AVKGFR**	1687.86	4+	677	270	1.80 × 10^−5^	3
**VVTNYNSAHDQNSNLLIEYFR/AVKGFR ^e^**	3156.59	4+	677	307	1.03 × 10^−5^	10
**NEFGEIQGDK/AVKGFR**	1795.90	3+	677	324	1.37 × 10^−8^	5
**VGQSMNMGSDFDVFAHITNNTAEEYVCR/AVKGFR ^e^**	3794.74	4+	677	481	1.99 × 10^−5^	5

^a^ Lysine and glutamine residues involved in isopeptide formation are underlined; ^b^ Position of the lysine and glutamine residue in the amino acid sequence of human tissue transglutaminase (TG2, P21980); ^c^ pLink2 E-value score <0.01 [28]; ^d^ Isopeptides identified in two or more independent MS^2^ scans are listed; ^e^ Isopeptide crosslinking sites already identified in Stamnaes et al. (2015) [18].

**Table 3:** Glutamine binding and deamidation sites in P1 (PQ_2_PQ_4_LPYPQ_9_PQ_11_LPY) involved in the formation of 22 isopeptides with different lysine residues of TG2. Modified sites identified with pLink2 are given in bold.

**(A) Isopeptides with Unambiguous Modification Sites (Localization Probability >75%)**													
**Position of K in TG2 ^a^**	***m*/*z***	**z**	**Score ^b^**	**Isopeptide Probability [%]**				**Deamidation Probability [%]**				**pLink2 Identification ^c^**	
				**Q_2_**	**Q_4_**	**Q_9_**	**Q_11_**	**Q_2_**	**Q_4_**	**Q_9_**	**Q_11_**	**E-value**	**No. MS^2^ Spectra**
**205**	818.77	3+	176.20	-	-	0.7	**99.3**	12.8	**87.2**	-	-	1.49 × 10^−9^	15
**380**	904.13	3+	153.63	-	-	0.4	**99.6**	-	**100**	-	-	1.37 × 10^−9^	8
**464**	929.48	3+	158.40	-	-	0.7	**99.3**	-	**100**	-	-	3.30 × 10^−6^	9
**590**	1051.22	3+	126.76	8.7	**91.3**	-	-	-	-	0.1	**99.9**	1.30 × 10^−11^	3
	1051.55	3+	72.29	1.1	7.8	**90.8**	0.3	**89.1**	10.9	0.4	**99.6**	1.95 × 10^−11^	5
**598**	854.46	3+	134.18	-	-	0.3	**99.7**	0.3	**99.7**	-	-	2.11 × 10^−11^	14
**600^c^**	1104.59	2+	165.83	-	-	-	100	-	100	-	-	-	-
**649**	1172.60	3+	64.65	-	0.1	2.1	**97.8**	10.5	**89.4**	-	0.1	2.00 × 10^−4^	7
**672**	980.85	3+	138.94	-	-	6.2	**93.8**	6.4	**93.6**	-	-	1.36 × 10^−9^	3
**677**	776.08	3+	84.29	8.1	**89.4**	0.5	2.1	0.2	2.1	0.9	96.8	2.85 × 10^−5^	3
**(B) Isopeptides with Ambiguous Modification Sites**													
**464**	697.61	4+	61.64	0.9	0.8	**91.7**	6.5	49.9	**50.1**	6.8	**93.2**	1.46 × 10^−3^	6
**468**	1005.49	3+	43.99	0.9	4.6	50.6	43.9	10.0	84.3	1.0	4.6	-	-
	1005.49	3+	40.73	0.2	2.2	19.3	78.3	41.8	55.2	0.8	2.2	-	-
**562**	1013.85	3+	53.26	-	0.2	11.5	**88.3**	29.9	**69.8**	0.1	0.2	1.36 × 10^−3^	2
**590**	1051.22	3+	158.30	-	-	63.0	37.0	-	100	-	-	-	-
	1051.22	3+	33.20	0.9	3.2	16.4	**79.5**	23.9	**66.7**	3.6	5.8	5.89 × 10^−15^	10
**600^d^**	694.03	3+	165.35	-	-	2.2	97.8	63.6	36.4	-	-	-	-
	694.36	3+	44.62	10.2	28.8	23.8	37.3	76.3	62.1	52.0	9.6	-	-
**(C) Isopeptides Identified with pLink2 ^d^**													
			**Crosslink**					**Deamidation**					
**30**	798.10	3+	Q_11_					Q_4_				1.20 × 10^-4^	3
	797.77	3+	Q_4_					-				7.62 × 10^-5^	3
**205**	818.77	3+	Q_9_					Q_4_				7.67 × 10^-3^	6
**429**	832.77	3+	Q_11_					Q_4_				1.95 × 10^-4^	4

^a^ Position of the lysine residue in the amino acid sequence of human tissue transglutaminase (TG2, P21980), corresponding to the following peptides: 30, EKLVVR; 205, FLKNAGR; 380, AIKEGDLSTK; 429, ISTKSVGR; 464, ANLHLNKLEAK; 468, LEAKEETGMAMR; 562, DCLTESNLIKVR; 590, DLYLENPEIKIR; 598, ILGEPKQK; ^c^ 600, QKR; ^d^ 600 QKRK; 649, TVEIPDPVEAGEEVKVR; 672, LVVNFESDKLK; 677, AVKGFR; ^b^ MaxQuant score calculated from the search against the α-gliadin fasta (P18573) for P1 carrying either TG2-modification, two different scores identify the same isopeptide with different binding sites within the model peptide; ^c^ pLink2 E-value <0.01 [28]; ^d^ not detected with MaxQuant.

**Table 4:** Glutamine binding and deamidation sites in P2 (VQ_2_GQ_4_GIIQ_8_PQ_10_Q_11_PAQ_14_L) involved in the formation of 33 isopeptides with different lysine residues of TG2. Modified sites identified with pLink2 are given in bold.

**(A) Isopeptides with Unambiguous Modification Sites (Localization Probability >75%)**																	
**Position of K in TG2 ^a^**	***m*/*z***	**z**	**Score ^b^**	**Isopeptide Probability [%]**						**Deamidation Probability [%]**						**pLink2 Identification ^c^**	
				**Q_2_**	**Q_4_**	**Q_8_**	**Q_10_**	**Q_11_**	**Q_14_**	**Q_2_**	**Q_4_**	**Q_8_**	**Q_10_**	**Q_11_**	**Q_14_**	**E-Value**	**No. MS^2^ Spectra**
**205**	798.76	3+	142.10	0.6	1.0	0.4	87.4	10.6	-	99.4	99.0	-	0.6	-	-	-	-
	798.43	3+	139.74	-	-	-	**99.6**	0.4	-	0.5	**99.5**	-	-	-	-	2.35 × 10^−6^	5
**380**	884.13	3+	125.61	**90.7**	8.1	1.2	-	-	-	8.3	**91.7**	0.2	**98.2**	1.6	-	9.80 × 10^−7^	2
	884.13	3+	128.46	-	-	0.1	**99.1**	0.8	-	0.4	**99.6**	-	-	-	-	8.31 × 10^−7^	3
**464**	682.12	4+	149.69	-	-	-	**99.8**	0.2	-	1.4	**98.6**	-	-	-	-	1.06 × 10^−6^	7
	682.36	4+	131.69	89.6	10.4	-	-	-	-	10.4	88.3	1.3	98.7	1.3	-	-	-
**590**	1031.22	3+	157.56	**93.6**	6.4	-	-	-	-	6.4	**93.6**	-	**98.3**	1.7	-	6.60 × 10^−11^	8
	1031.22	3+	126.32	10.5	**89.5**	-	-	-	-	**89.5**	10.5	0.1	**96.9**	3.1	-	1.61 × 10^−8^	8
	1031.54	3+	71.88	-	-	-	-	0.1	**99.9**	**100**	**99.9**	0.5	**85.3**	14.2	-	3.53 × 10^−9^	4
**598**	834.13	3+	126.32	-	0.9	14.7	**83.8**	0.5	-	0.6	**98.5**	-	0.9	-	-	3.65 × 10^−3^	2
	834.79	3+	75.46	98.9	1.1	-	-	-	-	1.1	97.4	2.5	87.8	11.6	99.6	-	-
**600**	1010.05	2+	232.88	-	-	-	100	-	-	0.1	99.9	-	-	-	-	-	-
	674.03	3+	139.19	99.2	0.8	-	-	-	-	0.8	99.2	-	93.3	6.7	-	-	-
**649**	1151.95	3+	52.82	-	-	-	0.2	1.9	97.9	-	-	-	-	-	-	-	-
	1152.60	3+	51.55	0.5	2.6	3.0	2.8	77.2	13.9	86.3	13.1	1.9	94.6	3.8	0.4	-	-
**672**	960.85	3+	67.02	96.7	3.1	0.2	-	-	-	3.1	95.7	1.5	95.9	3.8	-	-	-
**677**	756.08	3+	120.60	**93.0**	7.0	-	-	-	-	7.0	**92.9**	0.1	**96.3**	3.7	-	2.12 × 10^−5^	5
	756.08	3+	115.12	**98.5**	1.5	-	-	-	-	1.4	**98.5**	10.9	**89.0**	0.2	-	1.33 × 10^−6^	4
	755.75	3+	60.18	-	-	1.4	**76.6**	21.9	0.1	0.6	**99.0**	0.3	0.1	-	-	4.29 × 10^−5^	3
**(B) Isopeptides with Ambiguous Modification Sites**																	
**649**	1152.60	3+	78.53	**58.2**	14.9	26.0	0.8	0.1	-	35.0	**62.1**	3.0	**77.2**	22.7	0.1	9.03 × 10^−4^	3
	1152.93	3+	60.40	3.3	30.0	5.0	60.3	0.4	0.9	96.1	66.5	2.2	35.9	8.7	90.5	-	-
**(C) Isopeptides Identified with pLink2 ^d^**																	
			**Crosslink**							**Deamidation**							
**30**	778.10	3+	Q_2_							Q_4_; Q_10_						8.12 × 10^−5^	3
	778.10	3+	Q_4_							Q_2_; Q_10_						3.33 × 10^−5^	2
**380**	884.46	3+	Q_2_							Q_4_; Q_10_; Q_14_						1.59 × 10^−3^	4
**429**	812.77	3+	Q_10_							Q_2_; Q_4_						4.79 × 10^−5^	6
**464**	909.15	3+	Q_11_							Q_4_						5.29 × 10^−6^	2
	682.12	4+	Q_4_							Q_10_						4.42 × 10^−7^	5
**590**	1031.22	3+	Q_8_							Q_4_; Q_10_						2.46 × 10^−6^	3
**649**	1152.60	3+	Q_11_							Q_4_; Q_10_						1.07 × 10^−5^	4
	1152.60	3+	Q_8_							Q_4_; Q_10_						1.72 × 10^−6^	2
	1152.93	3+	Q_14_							Q_2_; Q_10_; Q_14_						2.12 × 10^−6^	2
**672**	960.52	3+	Q_10_							Q_4_						2.81 × 10^−4^	2
	960.52	3+	Q_4_							Q_10_						1.01 × 10^−3^	2

^a^ Position of the lysine residue in the amino acid sequence of human tissue transglutaminase (TG2, P21980), corresponding to the following peptides: 30, EKLVVR; 205, FLKNAGR; 380, AIKEGDLSTK; 429, ISTKSVGR; 464, ANLHLNKLEAK; 590, DLYLENPEIKIR; 598, ILGEPKQK; 600, QKR; 649, TVEIPDPVEAGEEVKVR; 672, LVVNFESDKLK; 677, AVKGFR; ^b^ MaxQuant score calculated from the search against the α-gliadin fasta (P18573) for P2 carrying either TG2-modification, two or three different scores identify the same isopeptide with different binding sites within the model peptide; ^c^ pLink2 E-value <0.01 [28]; ^d^ not detected with MaxQuant.

**Table 5:** Glutamine binding and deamidation sites in P3 (LQ_2_PQ_4_Q_5_PQ_7_Q_8_SFPQ_12_Q_13_Q_14_Q_15_PL) involved in the formation of 29 isopeptides with different lysine residues of TG2. Modified sites identified with pLink2 are given in bold.

**(A) Isopeptides with Unambiguous Modification Sites (Localization Probability >75%)**																							
**Position of K in TG2 ^a^**	***m*/*z***	**z**	**Score ^b^**	**Isopeptide Probability [%]**									**Deamidation Probability [%]**									**pLink2 Identification ^c^**	
				**Q_2_**	**Q_4_**	**Q_5_**	**Q_7_**	**Q_8_**	**Q_12_**	**Q_13_**	**Q_14_**	**Q_15_**	**Q_2_**	**Q_4_**	**Q_5_**	**Q_7_**	**Q_8_**	**Q_12_**	**Q_13_**	**Q_14_**	**Q_15_**	**E-value**	**No. MS^2^ Spectra**
**205**	937.47	3+	184.99	-	80.3	11.9	7.8	0.1	-	-	-	-	-	7.4	3.7	88.6	0.2	80.2	28.7	91.0	-	-	-
**590**	1169.93	3+	133.23	4.9	90.1	4.9	-	-	-	-	-	-	-	-	-	-	-	99.3	16.4	82.1	2.2	-	-
**600**	812.74	3+	150.76	-	83.8	12.4	3.2	0.5	-	-	-	-	-	2.2	4.1	81.6	12.1	86.1	33.7	80.2	-		
**649**	1291.31	3+	83.37	0.6	77.8	19.4	1.7	0.5	-	-	-	-	0.1	1.1	1.9	77.6	19.5	90.9	75.8	24.1	0.9	-	-
**677**	894.46	3+	140.22	0.3	87.4	11.9	0.3	-	-	-	-	-	-	-	-	-	-	98.4	16.8	84.6	0.2		
**(B) Isopeptides with Ambiguous Modification Sites**																							
**205**	937.46	3+	169.37	-	-	-	-	-	**76.3**	22.8	0.9	-	-	**92.8**	7.6	**24.3**	75.2	1.0	**2.2**	96.8	-	1.49 × 10^−5^	7
	937.46	3+	153.48	0.1	31.0	4.6	**63.8**	0.5	-	-	-	-	-	31.1	**34.5**	34.0	0.5	**73.6**	**31.2**	94.6	0.5	9.00 × 10^−5^	5
**380**	1022.85	3+	153.23	-	21.0	2.6	**76.2**	0.2	-	-	-	-	-	**32.4**	48.5	19.0	0.1	**82.7**	**29.5**	85.8	2.1	1.82 × 10^−4^	6
**464**	1048.20	3+	104.94	-	23.9	18.4	27.4	27.2	2.5	0.3	0.3	-	-	2.6	20.3	29.0	28.9	94.4	53.1	48.2	0.1	-	-
	786.64	4+	103.88	0.4	72.8	13.1	12.6	1.2	-	-	-	-	7.6	8.9	37.4	39.1	6.9	94.5	44.7	57.3	3.5	-	-
	786.64	4+	71.55	-	0.1	1.2	**76.3**	19.6	0.8	0.5	0.5	0.1	30.3	**49.6**	20.5	21.5	78.9	**37.8**	**75.1**	70.9	15.3	4.86 × 10^−4^	4
**590**	1169.60	3+	147.56	-	12.4	12.4	**67.5**	7.7	-	-	-	-	0.2	53.2	21.9	21.1	3.6	**60.7**	84.4	**53.8**	1.2	4.70 × 10^−9^	2
**598**	973.17	3+	148.70	-	9.2	1.5	44.6	44.6	-	-	-	-	-	38.4	51.4	5.1	5.1	96.4	98.7	4.8	-	-	-
	973.17	3+	140.77	-	30.6	6.2	**60.6**	2.5	0.1	0.1	-	-	-	**32.9**	32.4	33.4	1.5	**87.0**	**95.0**	17.8	-	1.15 × 10^−3^	4
	972.84	3+	125.50	-	45.6	**45.6**	8.6	0.2	-	-	-	-	-	-	-	-	-	**48.7**	76.9	**70.7**	3.6	8.06 × 10^−4^	2
**600**	812.74	3+	124.50	-	14.4	81.1	3.3	1.2	-	-	-	-	0.1	78.7	11.2	7.9	2.1	89.7	55.1	55.1	0.1	-	-
**649**	1291.64	3+	78.33	-	0.4	3.4	21.1	**70.9**	0.4	0.2	-	-	0.6	**49.0**	54.1	72.3	23.5	**82.3**	50.7	**52.7**	14.8	2.85 × 10^−5^	2
	1291.64	3+	78.33	-	0.4	3.4	21.1	**70.9**	0.4	0.2	-	-	0.6	**49.0**	54.1	72.3	23.5	**82.3**	**50.7**	52.7	14.8	1.39 × 10^−5^	2
	1291.64	3+	60.54	1.4	**68.1**	27.5	2.3	0.5	0.1	-	-	-	-	0.1	0.2	0.6	5.6	**71.3**	36.5	**53.9**	31.6	2.63 × 10^−6^	3
**677**	894.79	3+	178.14	-	6.4	1.0	89.3	3.4	-	-	-	-	-	36.5	56.6	6.4	0.4	99.8	99.8	0.4	-	-	-
	894.46	3+	120.59	-	5.1	70.1	23.3	1.2	0.1	0.1	0.1	-	-	0.1	0.1	-	-	98.8	49.4	49.4	2.1	-	-
	894.45	3+	51.70	-	0.1	0.1	0.4	0.4	24.7	24.7	24.7	24.8	0.3	47.1	52.9	49.0	54.9	74.0	74.0	74.0	73.8	-	-
**(C) Isopeptides identified with pLink2 ^d^**																							
			**Crosslink**										**Deamidation**										
**30**	916.48	3+	Q_4_										Q_12_; Q_14_									4.14 × 10^−4^	3
**380**	1022.52	3+	Q_5_										Q_12_; Q_13_									6.95 × 10^−5^	3
**429**	951.15	3+	Q_12_										Q_4_; Q_14_									6.77 × 10^−5^	2
**464**	786.15	4+	Q_12_										Q_4_; Q_13_									3.16 × 10^−4^	6
	786.64	4+	Q_12_										Q_2_; Q_10_; Q_14_									3.39 × 10^−4^	3
**590**	1169.60	3+	Q_12_										Q_4_; Q_7_; Q_13_									1.21 × 10^−6^	2
	1169.60	3+	Q_7_										Q_12_; Q_14_									5.18 × 10^−9^	2

^a^ Position of the lysine residue in the amino acid sequence of human tissue transglutaminase (TG2, P21980), corresponding to the following peptides: 30, EKLVVR; 205, FLKNAGR; 380, AIKEGDLSTK; 429, ISTKSVGR; 464, ANLHLNKLEAK; 590, DLYLENPEIKIR; 598, ILGEPKQK; 600, QKR; 649, TVEIPDPVEAGEEVKVR; 672, LVVNFESDKLK; 677, AVKGFR; ^b^ MaxQuant score calculated from the search against the α-gliadin fasta (P18573) for P3 carrying either TG2-modification, two or three different scores identify the same isopeptide with different binding sites within the model peptides; ^c^ pLink2 E-value <0.01 [28]; ^d^ not detected with MaxQuant.

## Data Availability

The mass spectrometry proteomics data have been deposited to the ProteomeXchange Consortium (http://proteomecentral.proteomexchange.org) via the PRIDE [35] partner repository with the dataset identifier PXD014067. The extracted precursor ion chromatograms of selected isopeptides analyzed with Skyline were made publicly available on Panorama Public (https://panoramaweb.org/KwmI1z.url).

## Supplementary Materials

The following are available online at https://www.mdpi.com/2072-6643/11/10/2263/s1, Figure S1: Reciprocal search workflow to identify isopeptides with MaxQuant and Skyline, Figure S2: Sequence coverage of TG2, Figure S3: MS/MS spectra of isopeptides between human tissue transglutaminase (TG2) and PepQ (PFPQPQLPY) or its deamidated form PepE, Figure S4: Differentiation of the reactive sites of TG2, Table S1: TG2 peptides containing lysine residues identified as isopeptide crosslinking sites, Table S2: Alignment of the crosslinking sites of TG2 sorted by most preferred to least preferred in the model system.
