# Supplementary material for: Comprehensive Detection of Isopeptides between Human Tissue Transglutaminase and Gluten Peptides
Source: Nutrients. 2019 Sep 20;11(10):2263. doi: 10.3390/nu11102263 (PMC6835481; doi:10.3390/nu11102263)
Supplement: Supplementary file 1 [file nutrients-11-02263-s001.zip › supplM/isopeptides_fig_s1.pdf]

Supplementary Figure S1

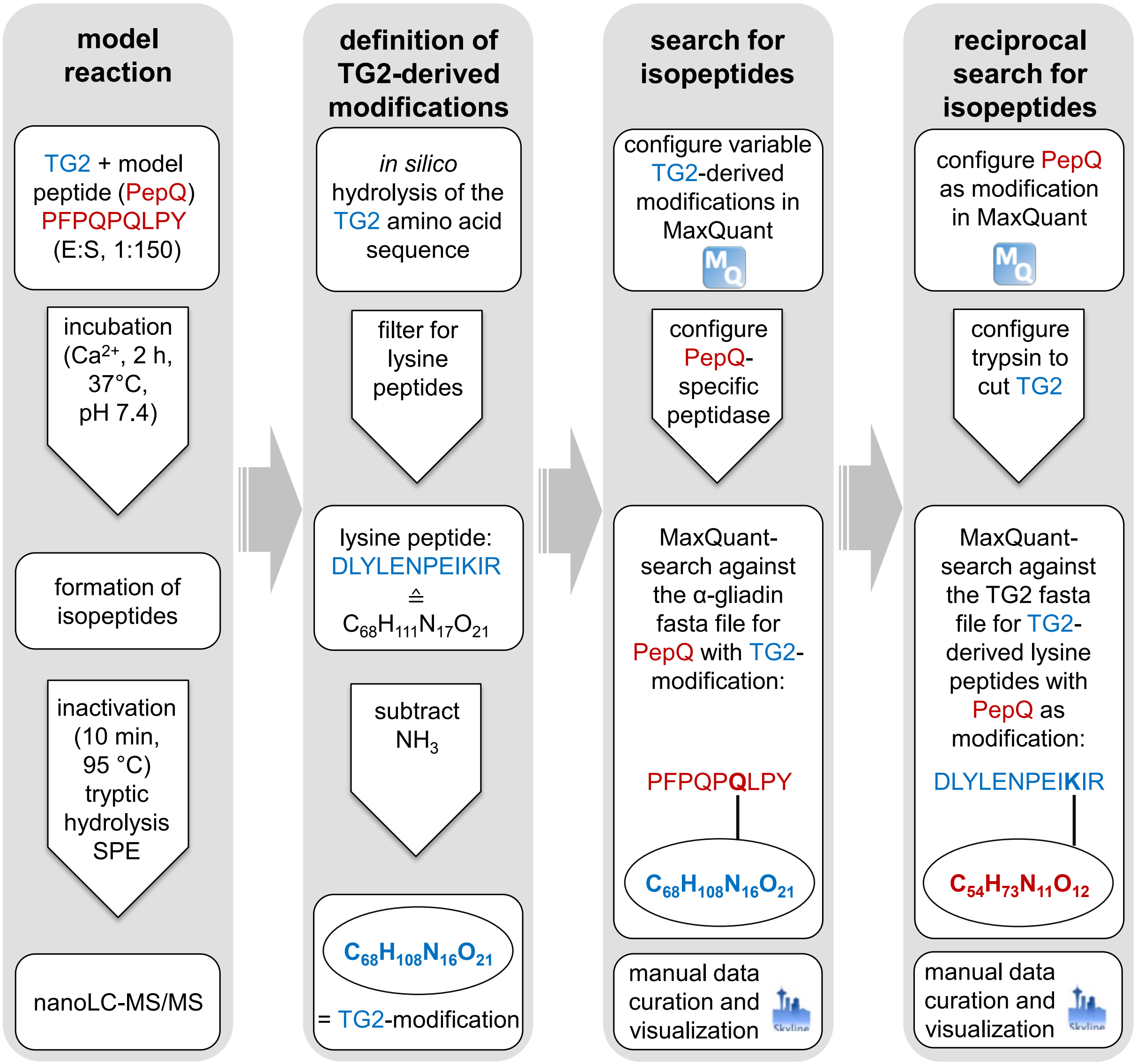

Reciprocal search workflow to identify isopeptides with MaxQuant and Skyline. The workflow consists of four steps: (1) model reaction between TG2 and gluten peptide(s) followed by tryptic hydrolysis, clean-up and untargeted nLC-MS/MS analysis, (2) definition of tryptic TG2-derived lysine peptides as potential isopeptide modification sites, (3) the search for isopeptides by configuring the TG2-modifications in MaxQuant and search against the α-gliadin fasta file and (4) the reciprocal search for isopeptides by configuring the gluten peptide(s) as modification in MaxQuant and search against the TG2 fasta file.
