# Supplementary material for: Comprehensive Detection of Isopeptides between Human Tissue Transglutaminase and Gluten Peptides
Source: Nutrients. 2019 Sep 20;11(10):2263. doi: 10.3390/nu11102263 (PMC6835481; doi:10.3390/nu11102263)
Supplement: Supplementary file 1 [file nutrients-11-02263-s001.zip › supplM/isopeptides_fig_s2.pdf]

```
        10         20         30         40         50
MAEELVLERC DLELETNGRD HHTADLCREK LVVRRGQPFW LTLHFEGRNY
        60         70         80         90        100
EASVDSLTFS VVTGPAPSQE AGTKARFPLR DAVEEGDWTA TVVDQQDCTL
       110        120        130        140        150
SLQLTTPANA PIGLYRLSLE ASTGYQGSSF VLGHFILLFN AWCPADAVYL
       160        170        180        190        200
DSEEERQEYV LTQQGFIYQG SAKFIKNIPW NFGQFEDGIL DICLILLDVN
       210        220        230        240        250
PKFLKNAGRD CSRRSSPVYV GRVVSGMVNC NDDQGVLLGR WDNNYGDGVS
       260        270        280        290        300
PMSWIGSVDI LRRWKNHGCQ RVKYGQCWVF AAVACTVLRC LGIPTRVVTN
       310        320        330        340        350
YNSAHDQNSN LLIEYFRNEF GEIQGDKSEM IWNFHCWVES WMTRPDLQPG
       360        370        380        390        400
YEGWQALDPT PQEKSEGTYC CGPVPVRAIK EGDLSTKYDA PFVFAEVNAD
       410        420        430        440        450
VVDWIQQDDG SVHKSINRSL IVGLKISTKS VGRDEREDIT HTYKYPEGSS
       460        470        480        490        500
EEREAFTRAN HLNKLAEKEE TGMAMRIRVG QSMNMGSFD VFAHITNNTA
       510        520        530        540        550
EEYVCRLLLC ARTVSYNGIL GPECGTKYLL NLNLEPFSEK SVPLCILYEK
       560        570        580        590        600
YRDCLTESNL IKVRALLVEP VINSYLLAER DLYLENPEIK IRILGEPKQK
       610        620        630        640        650
RKLVAEVSLQ NPLPVALEGC TFTVEGAGLT EEQKTVEIPD PVEAGEEVKV
       660        670        680
RMDLLPLHMG LHKLVVNFES DKLKAVKGFR NVIIGPA
```

Supplementary Figure S2.

Sequence coverage of TG2. The analysis of all identified peptides of human tissue transglutaminase (TG2) resulted in a sequence coverage of 79.6 % (UniProtKB accession No. P21980).
