# Supplementary material for: Comprehensive Detection of Isopeptides between Human Tissue Transglutaminase and Gluten Peptides
Source: Nutrients. 2019 Sep 20;11(10):2263. doi: 10.3390/nu11102263 (PMC6835481; doi:10.3390/nu11102263)
Supplement: Supplementary file 1 [file nutrients-11-02263-s001.zip › supplM/isopeptides_fig_s3_new.pdf]

A

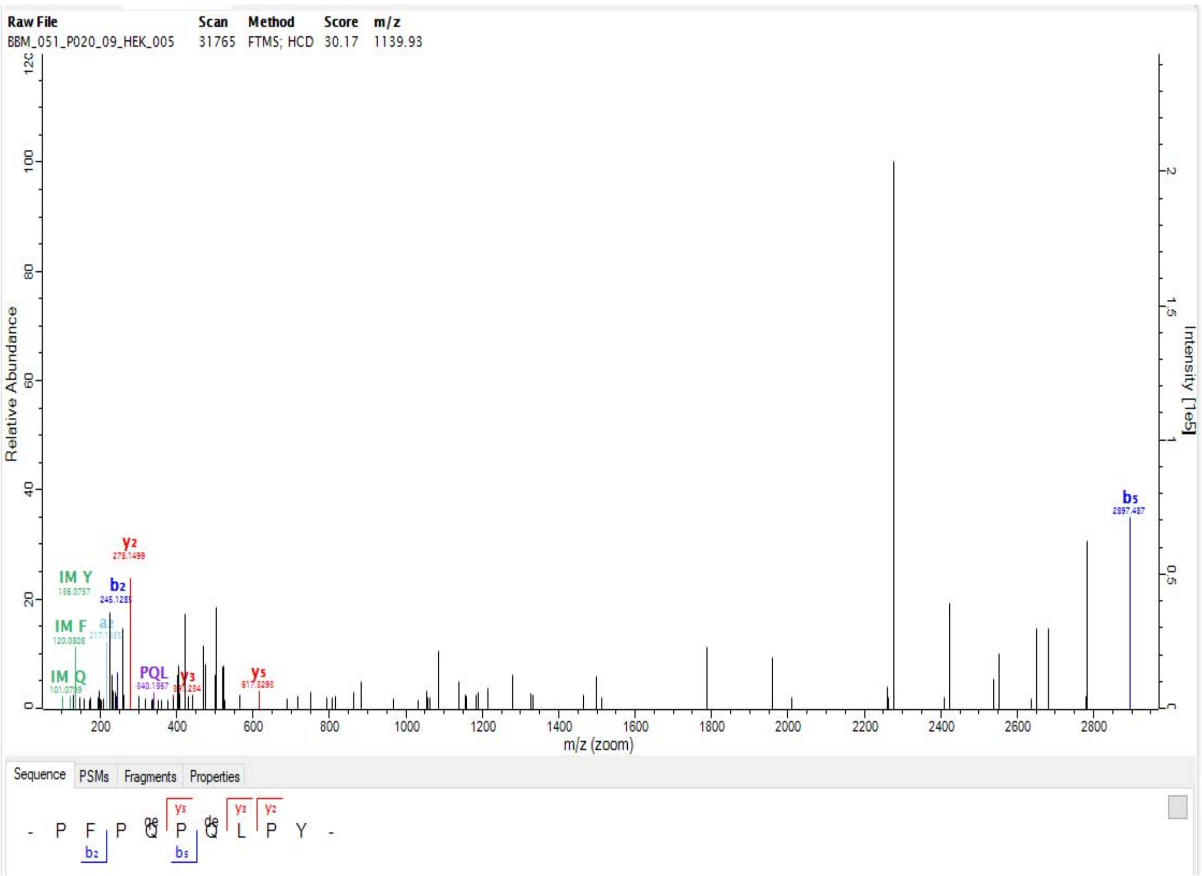
Raw File
BBM_051_P020_09_HEK_005
Scan
31765
Method
FTMS; HCD
Score
30.17
m/z
1139.93
Relative Abundance
Intensity [1e5]
m/z (zoom)
IM Y
IM F
IM Q
y2
b2
PQL
y3
y5
b5
Sequence
PSMs
Fragments
Properties
- P F P Q P Q L P Y -

B

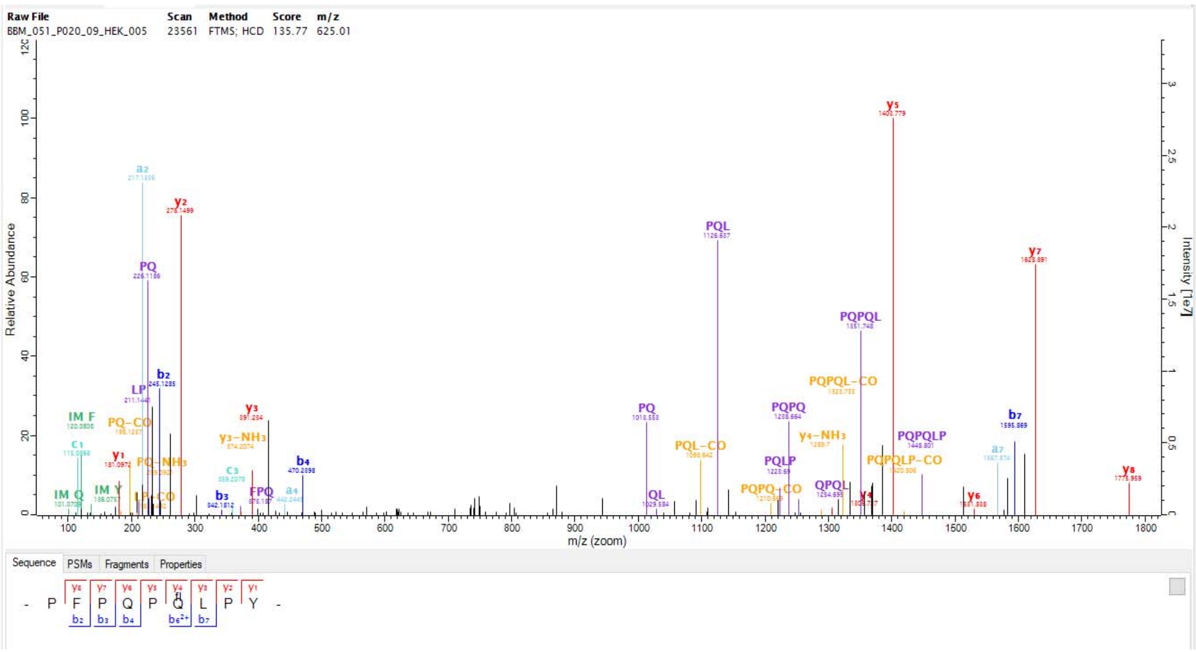
Raw File
BBM_051_P020_09_HEK_005
Scan
23561
Method
FTMS; HCD
Score
135.77
m/z
625.01
Relative Abundance
Intensity [1e7]
m/z (zoom)
a2
y2
PQ
b2
LP
IM F
PQ-CO
c1
y1
PQ-NH3
IM Q
IM Y
y3
y3-NH3
b4
c3
b3
FPQ
a4
PQL
PQ
QL
PQL-CO
PQPQL
PQPQL-CO
PQPQ
y4-NH3
PQLP
PQPQ-CO
QPQL
PQPQLP
PQPQLP-CO
y5
y7
b7
a7
y6
y8
Sequence
PSMs
Fragments
Properties
- P F P Q P Q L P Y -

C

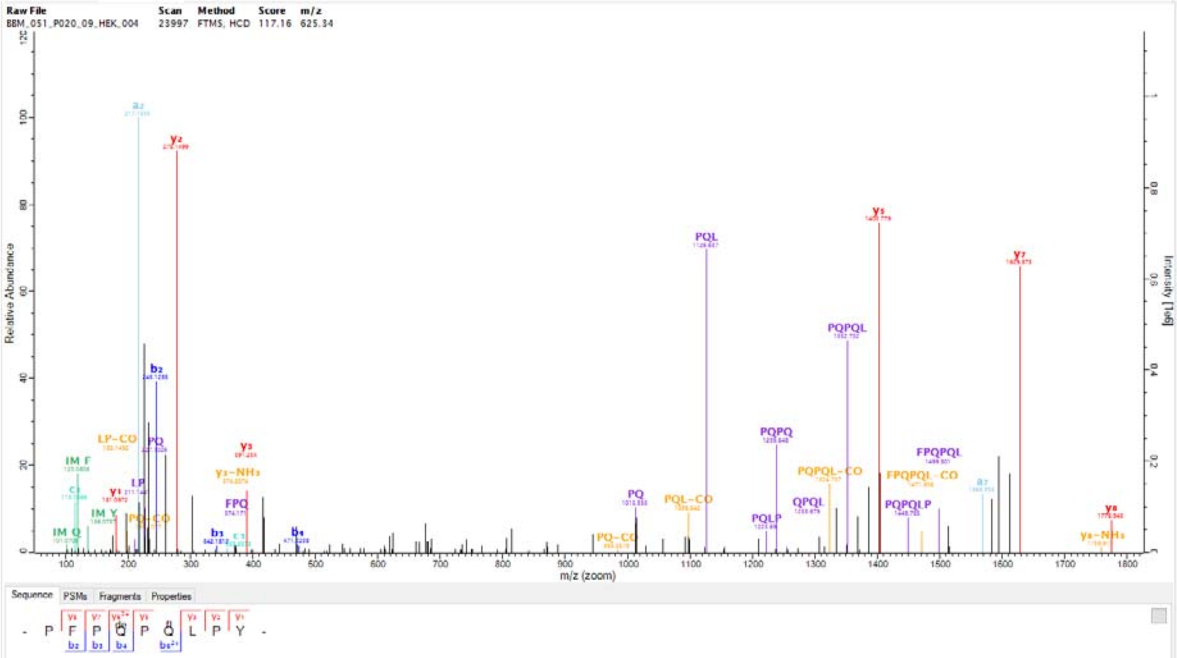
Raw File
BBM_051_P020_09_HEK_004
Scan
23997
Method
FTMS; HCD
Score
117.16
m/z
625.34
Relative Abundance
Intensity [1e6]
m/z (zoom)
Sequence
PSMs
Fragments
Properties
PQL
PQPQL
PQPQ
FPQPQL
FPQPQL-CO
PQPQL-CO
QPQL
PQPQLP
PQLP
PQ
PQL-CO
PQ-CO
LP-CO
IM F
IM Y
IM Q
LP
FPQ
- P F P Q P Q L P Y -

D

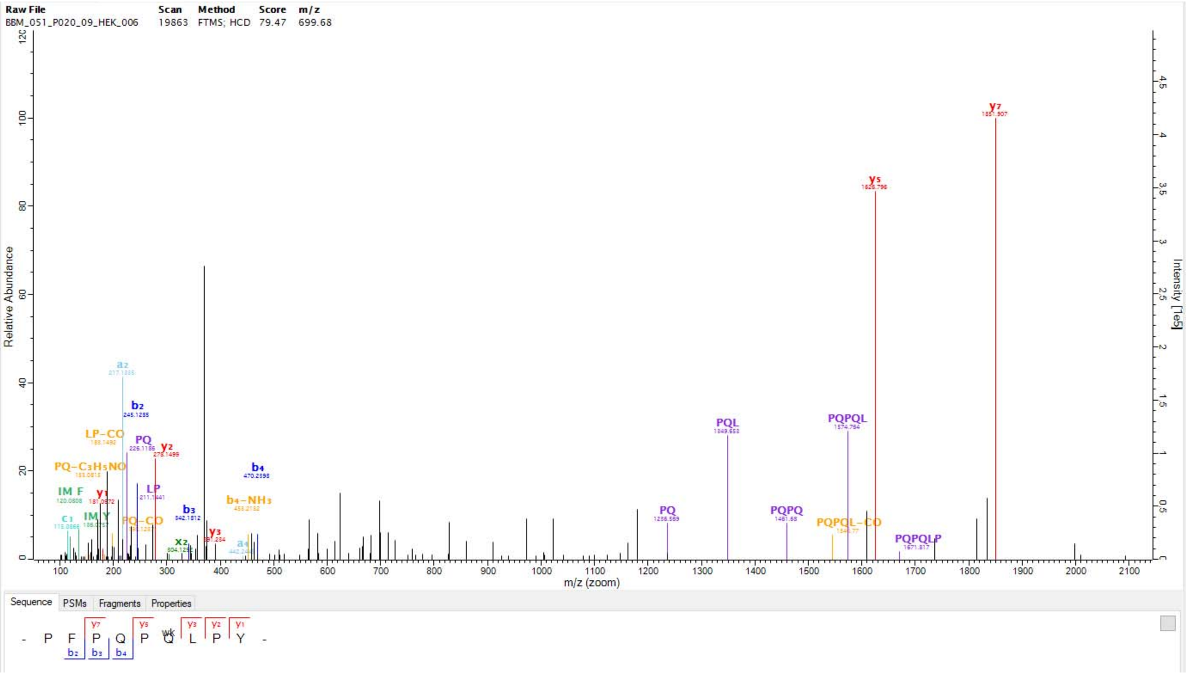
Raw File
BBM_051_P020_09_HEK_006
Scan
19863
Method
FTMS; HCD
Score
79.47
m/z
699.68
Relative Abundance
Intensity [1e5]
m/z (zoom)
Sequence
PSMs
Fragments
Properties
PQL
PQPQL
PQPQ
PQPQL-CO
PQPQLP
PQ
LP-CO
PQ-C3H5NO
IM F
IM Y
LP
PQ-CO
b4-NH3
- P F P Q P Q L P Y -

E

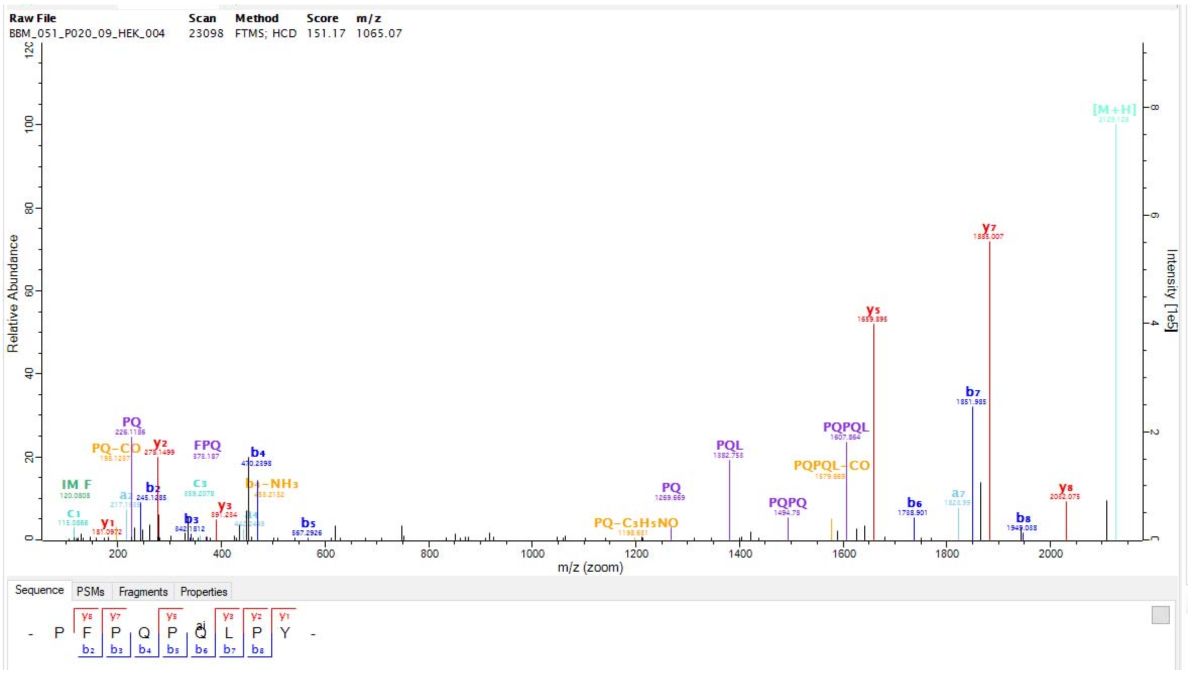
Raw File
BBM_051_P020_09_HEK_004
Scan
23098
Method
FTMS; HCD
Score
151.17
m/z
1065.07
Relative Abundance
Intensity [1e5]
m/z (zoom)
Sequence
PSMs
Fragments
Properties

F

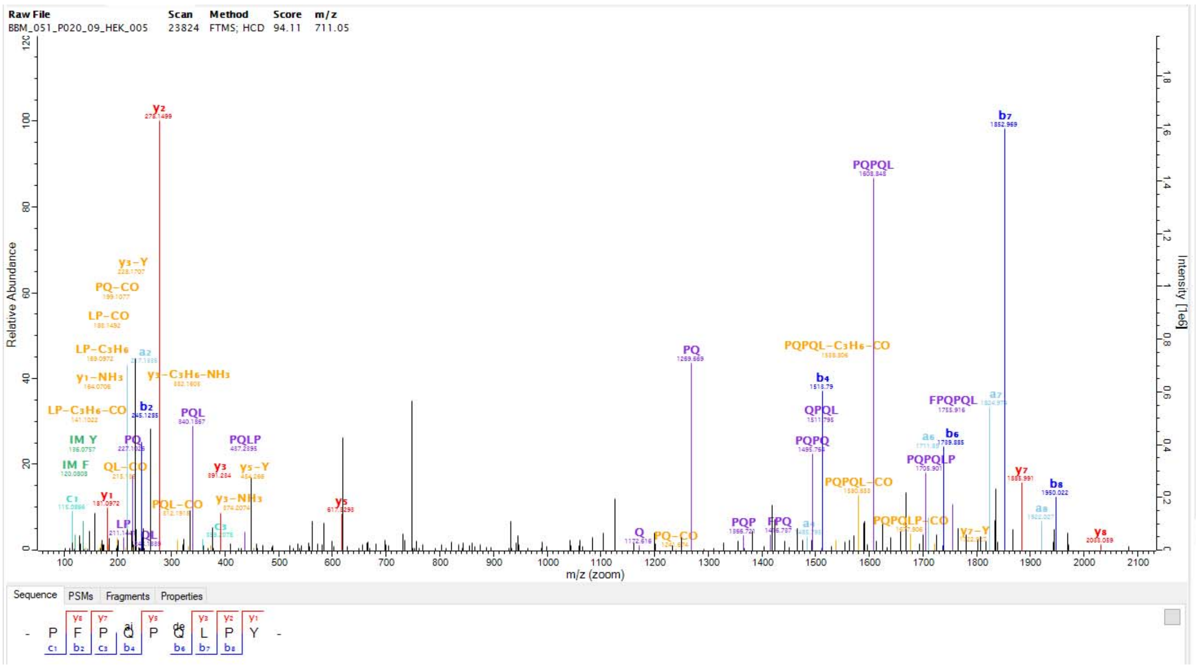
Raw File
BBM_051_P020_09_HEK_005
Scan
23824
Method
FTMS; HCD
Score
94.11
m/z
711.05
Relative Abundance
Intensity [1e6]
m/z (zoom)
Sequence
PSMs
Fragments
Properties

G

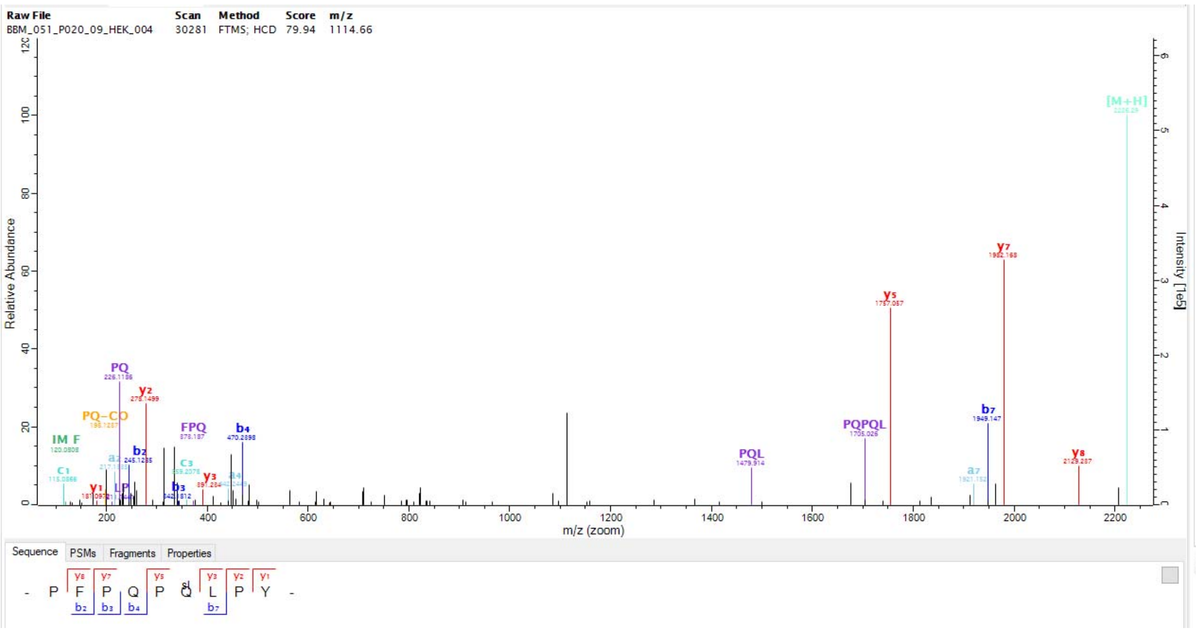
Raw File Scan Method Score m/z
BBM_051_P020_09_HEK_004 30281 FTMS; HCD 79.94 1114.66
Relative Abundance
m/z (zoom)
Intensity [1e5]
Sequence PSMs Fragments Properties

H

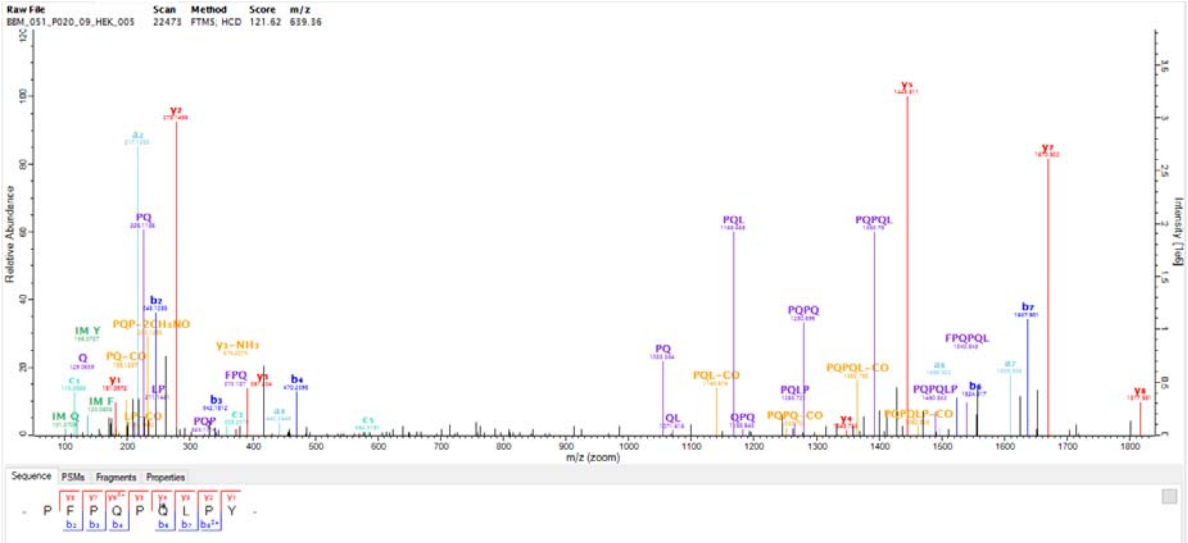
Raw File Scan Method Score m/z
BBM_051_P020_09_HEK_005 22473 FTMS; HCD 121.62 639.36
Relative Abundance
m/z (zoom)
Intensity [1e6]
Sequence PSMs Fragments Properties

I

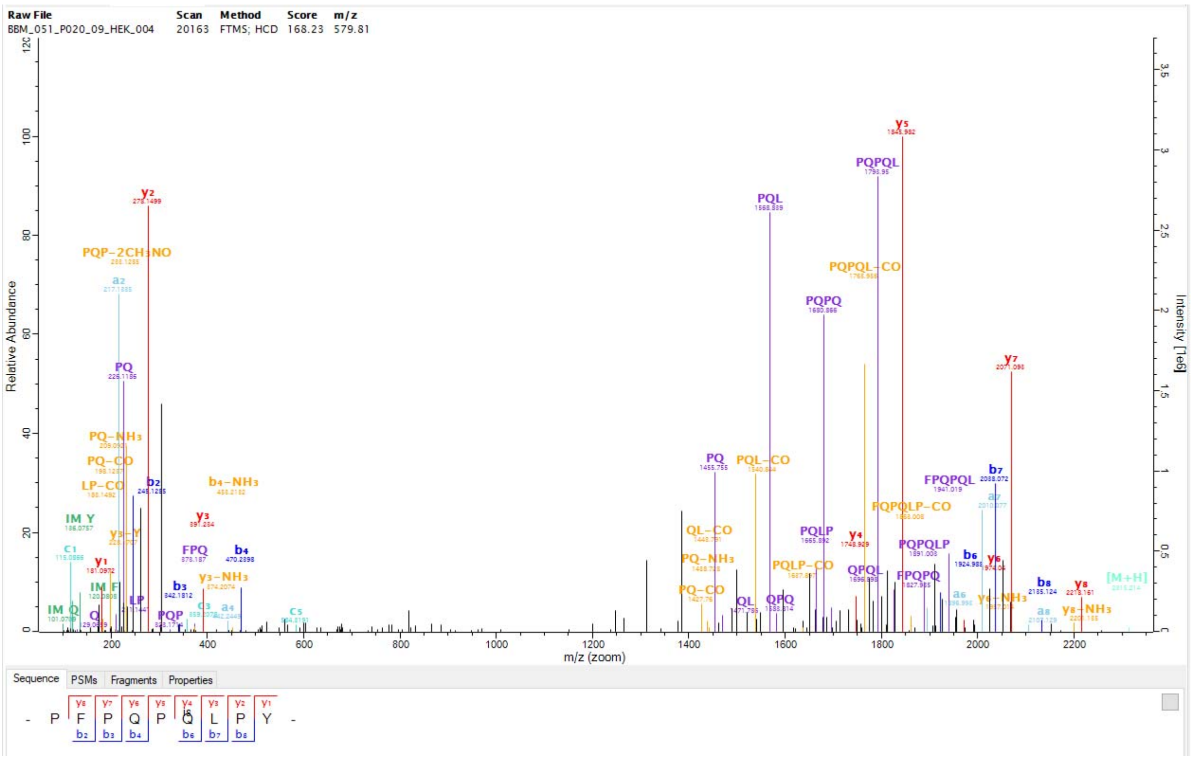
Raw File Scan Method Score m/z
BBM_051_P020_09_HEK_004 20163 FTMS; HCD 168.23 579.81
Relative Abundance
Intensity [1e6]
m/z (zoom)
Sequence PSMs Fragments Properties
- P F P Q P Q L P Y -

J

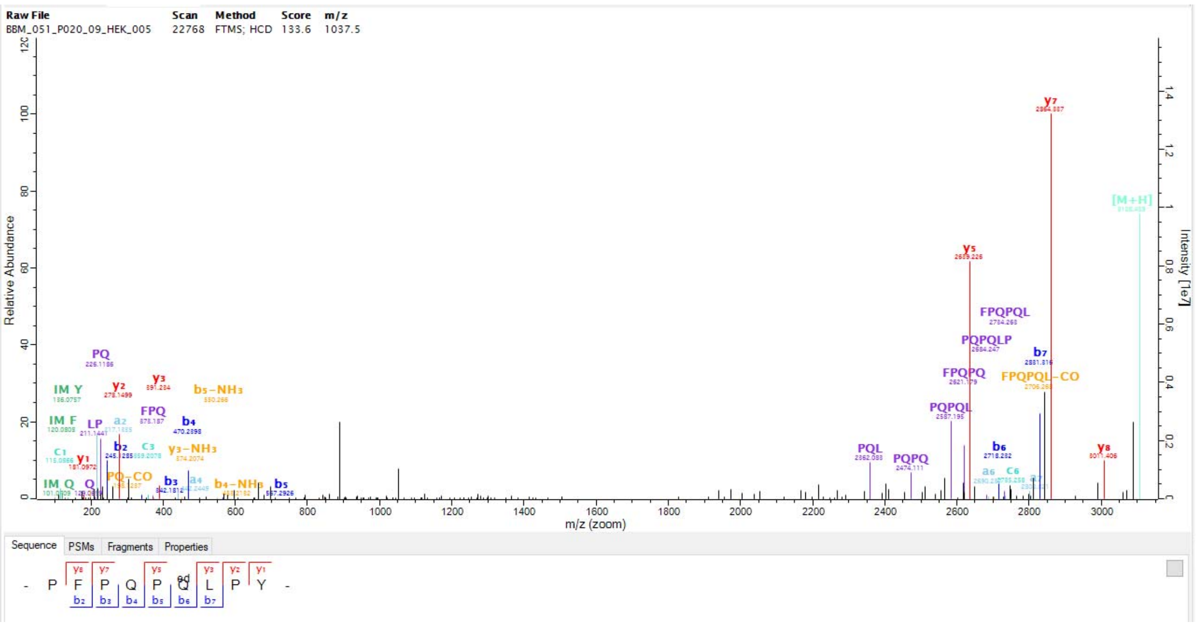
Raw File Scan Method Score m/z
BBM_051_P020_09_HEK_005 22768 FTMS; HCD 133.6 1037.5
Relative Abundance
Intensity [1e7]
m/z (zoom)
Sequence PSMs Fragments Properties
- P F P Q P Q L P Y -

K

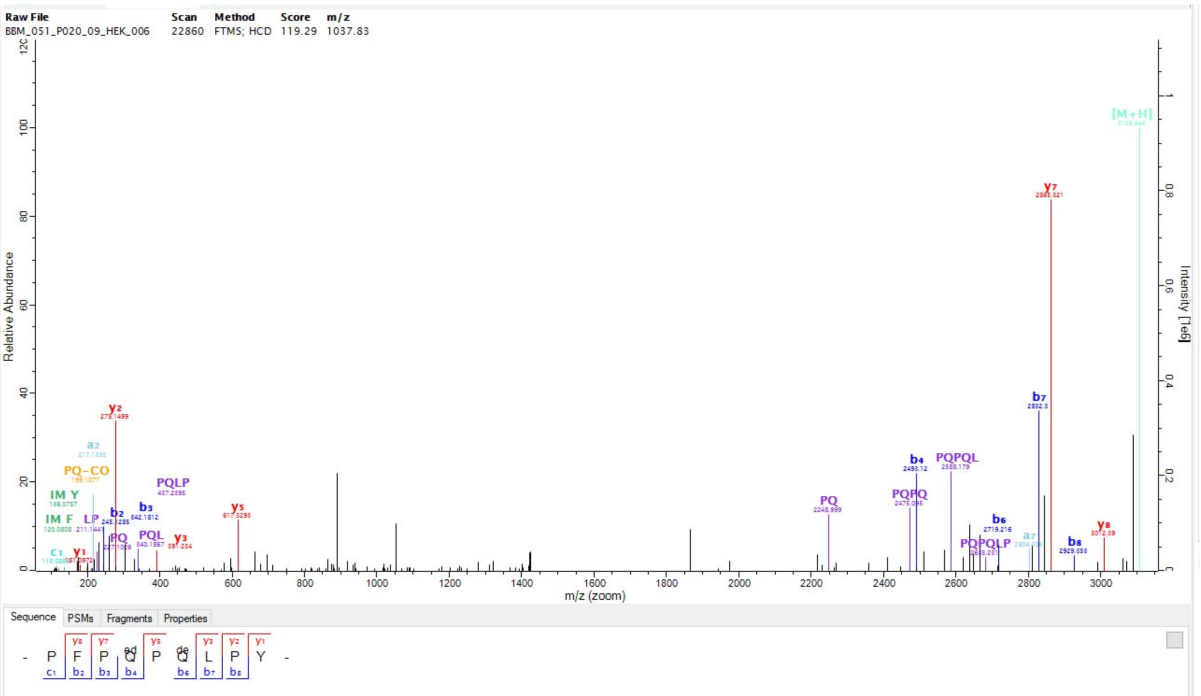

Raw File
BBM_051_P020_09_HEK_006
Scan
22860
Method
FTMS; HCD
Score
119.29
m/z
1037.83
Relative Abundance
Intensity [1e6]
m/z (zoom)
[M+H]
y7
b7
PQPQL
b4
PQPQ
PQ
b6
PQPQLP
a7
b8
y8
y2
a2
PQ-CO
IM Y
IM F
LP
b2
b3
PQLP
PQL
y5
y3
y1
c1
Sequence
PSMs
Fragments
Properties
- P F P Q P Q L P Y -


L

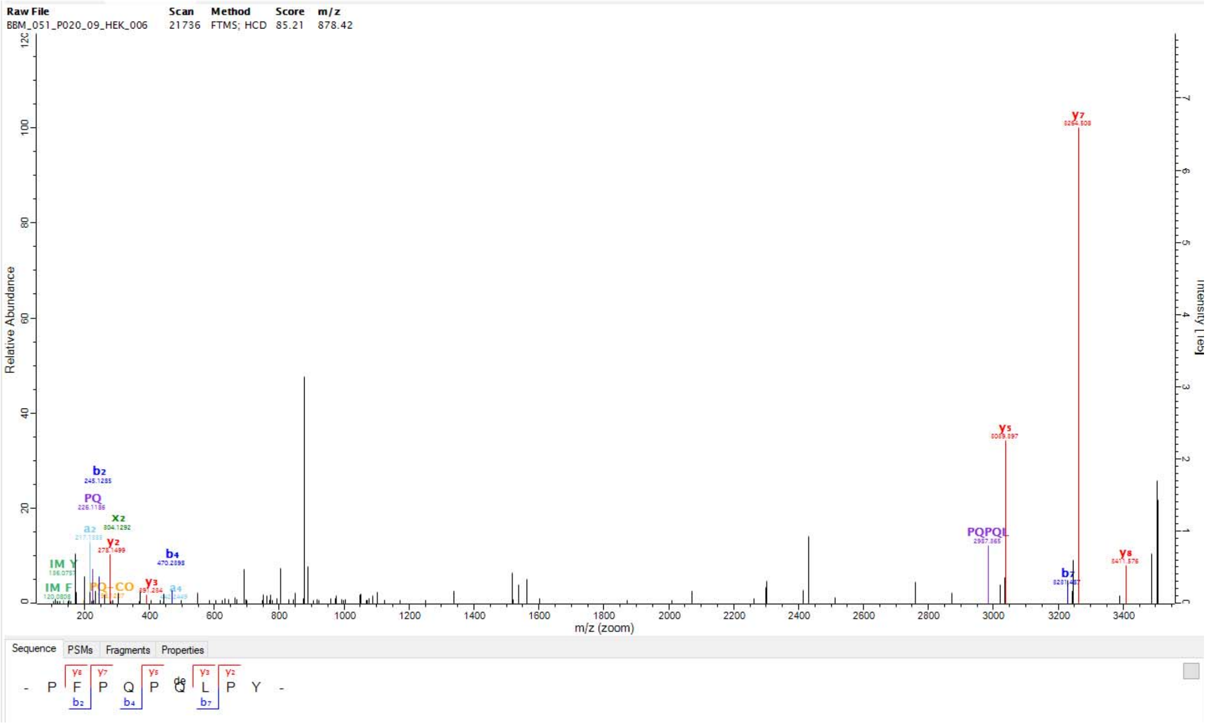

Raw File
BBM_051_P020_09_HEK_006
Scan
21736
Method
FTMS; HCD
Score
85.21
m/z
878.42
Relative Abundance
Intensity [1e6]
m/z (zoom)
y7
y5
PQPQL
b7
y8
b2
PQ
x2
a2
y2
IM Y
IM F
PQ-CO
y3
b4
a4
Sequence
PSMs
Fragments
Properties
- P F P Q P Q L P Y -

M

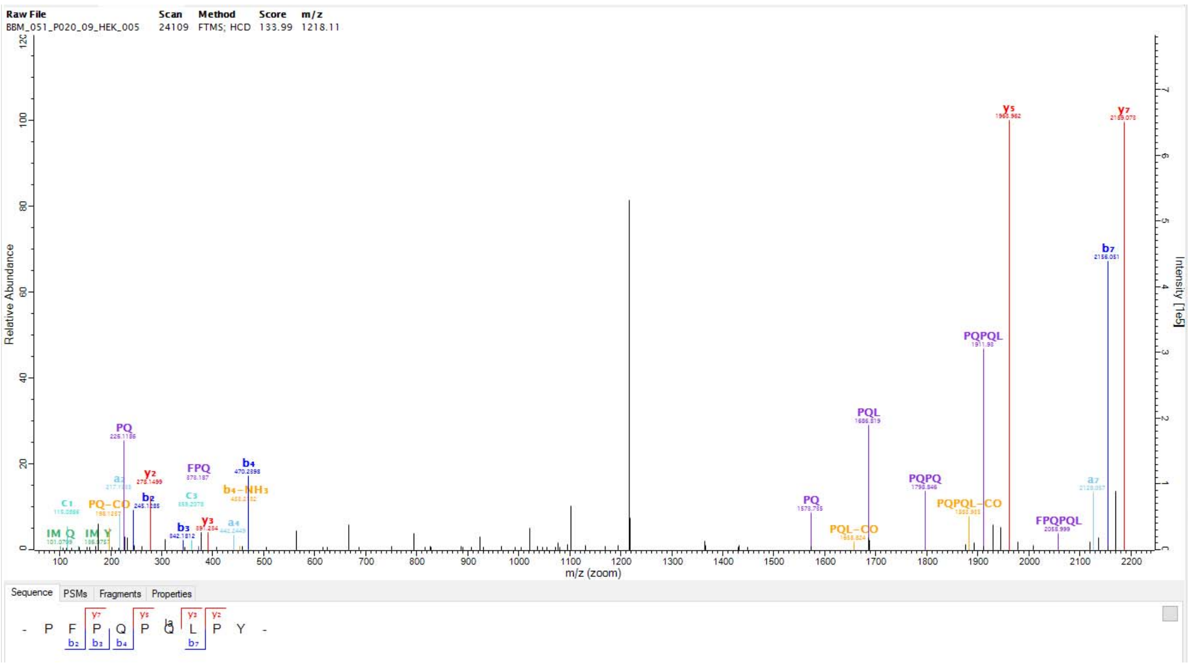

Raw File
BBM_051_P020_09_HEK_005
Scan
24109
Method
FTMS; HCD
Score
133.99
m/z
1218.11
Relative Abundance
Intensity [1e5]
m/z (zoom)
y5
y7
b7
PQPQL
PQL
PQPQ
PQPQL-CO
FPQPQL
PQL-CO
PQ
a7
PQ
FPQ
b4
b4-NH3
y2
b2
a2
C1
PQ-CO
IM Q
IM Y
c3
b3
y3
a4
Sequence
PSMs
Fragments
Properties
- P F P Q P Q L P Y -


N

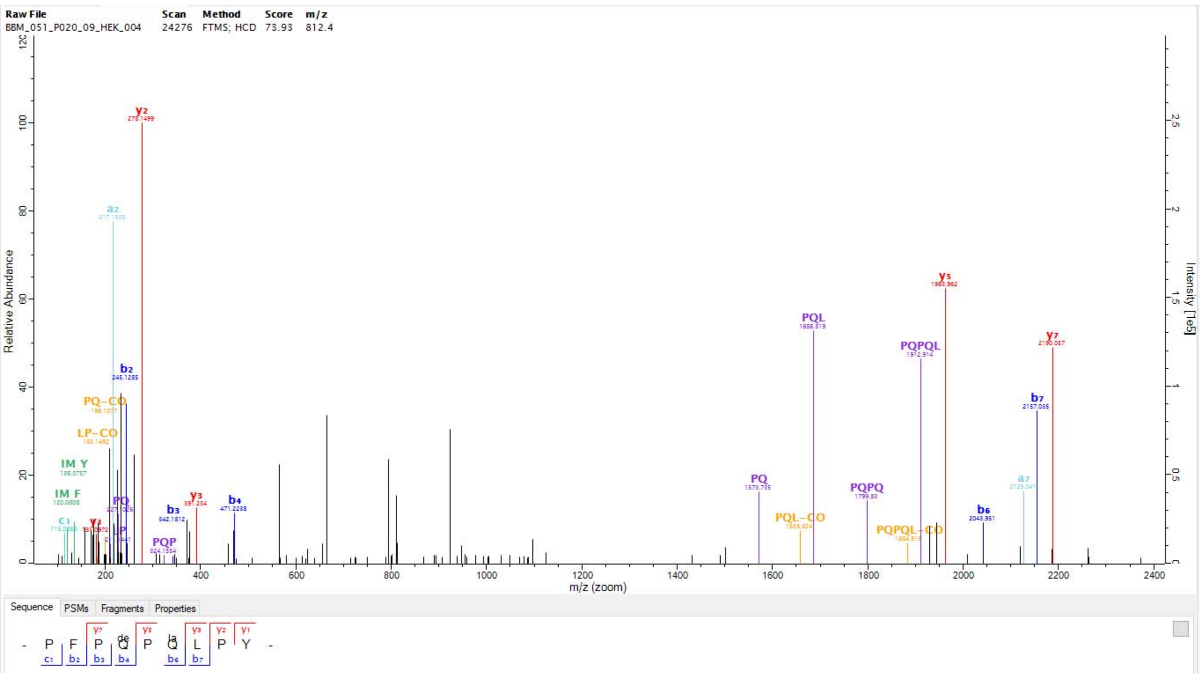

Raw File
BBM_051_P020_09_HEK_004
Scan
24276
Method
FTMS; HCD
Score
73.93
m/z
812.4
Relative Abundance
Intensity [1e5]
m/z (zoom)
y2
a2
b2
PQ-CO
LP-CO
IM Y
IM F
C1
PQ
b3
y3
b4
PQP
PQ
PQL
PQPQL
PQPQ
PQL-CO
PQPQL-CO
y5
y7
b7
b6
a7
Sequence
PSMs
Fragments
Properties
- P F P Q P Q L P Y -

O

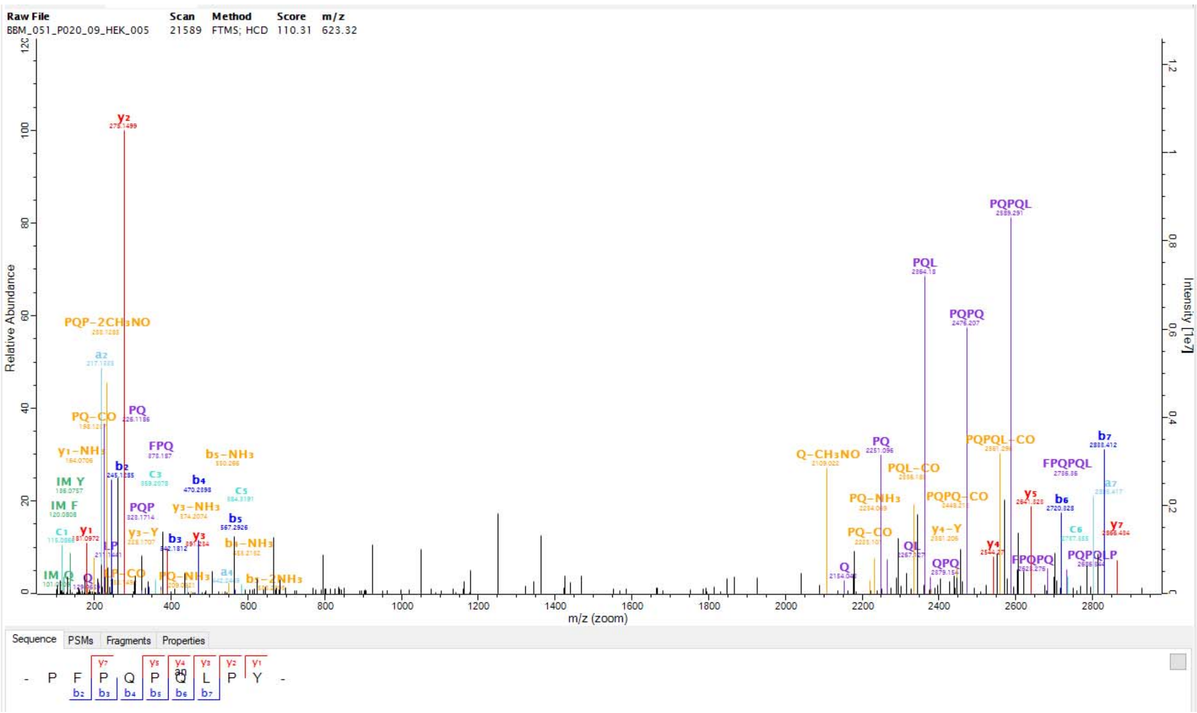
Raw File BBM_051_P020_09_HEK_005
Scan 21589
Method FTMS; HCD
Score 110.31
m/z 623.32
Relative Abundance
Intensity [1e7]
m/z (zoom)
Sequence
PSMs
Fragments
Properties
- P F P Q P Q L P Y -

P

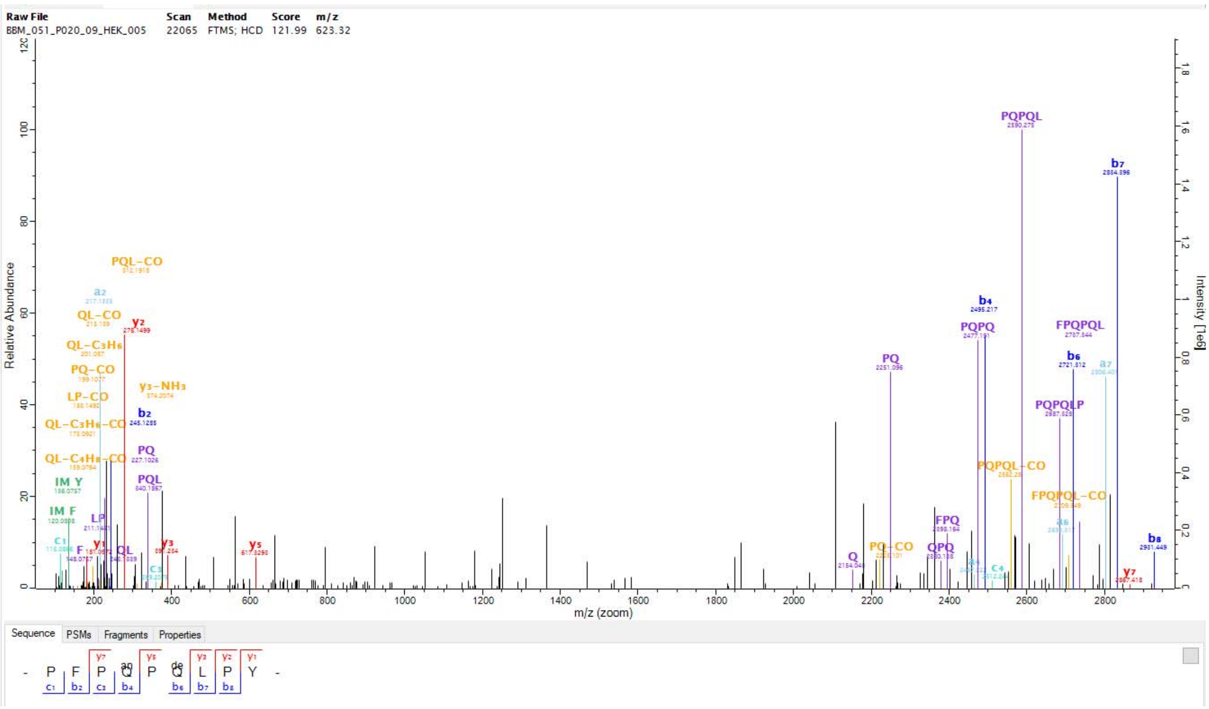
Raw File BBM_051_P020_09_HEK_005
Scan 22065
Method FTMS; HCD
Score 121.99
m/z 623.32
Relative Abundance
Intensity [1e6]
m/z (zoom)
Sequence
PSMs
Fragments
Properties
- P F P Q P Q L P Y -

Q

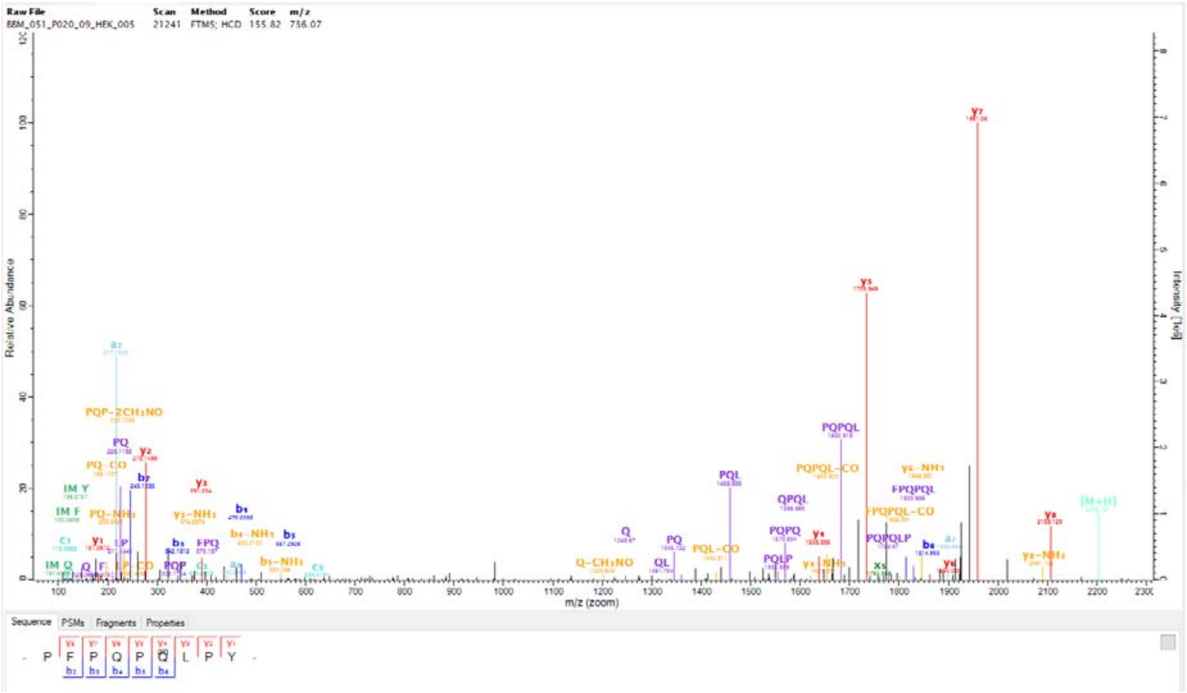
Raw File Scan Method Score m/z
BBM_051_P020_09_HEK_005 21241 FTMS; HCD 155.82 736.07
Relative Abundance
Intensity [1e5]
m/z (zoom)
Sequence PSMs Fragments Properties
- P F P Q P Q L P Y -

R

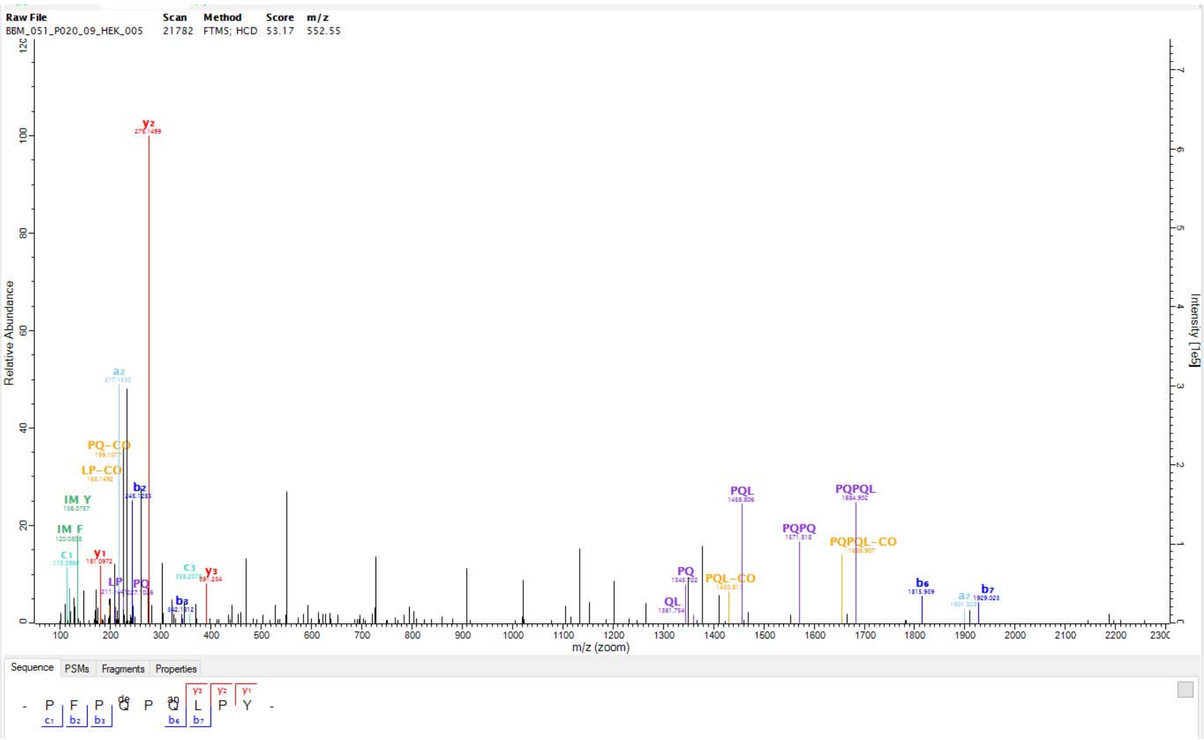
Raw File Scan Method Score m/z
BBM_051_P020_09_HEK_005 21782 FTMS; HCD 53.17 552.55
Relative Abundance
Intensity [1e5]
m/z (zoom)
Sequence PSMs Fragments Properties
- P F P Q P Q L P Y -

S

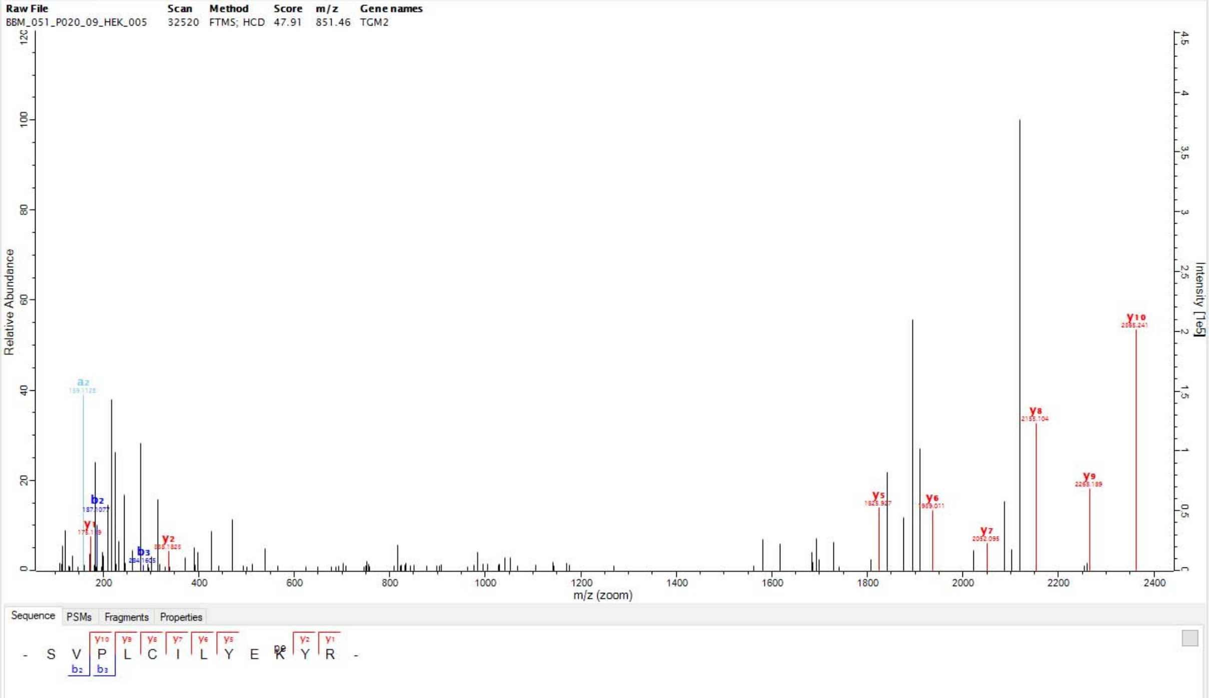
Raw File Scan Method Score m/z Gene names
BBM_051_P020_09_HEK_005 32520 FTMS; HCD 47.91 851.46 TGM2
Relative Abundance
Intensity [1e5]
m/z (zoom)
Sequence PSMs Fragments Properties
- S V P L C I L Y E K Y R -

T

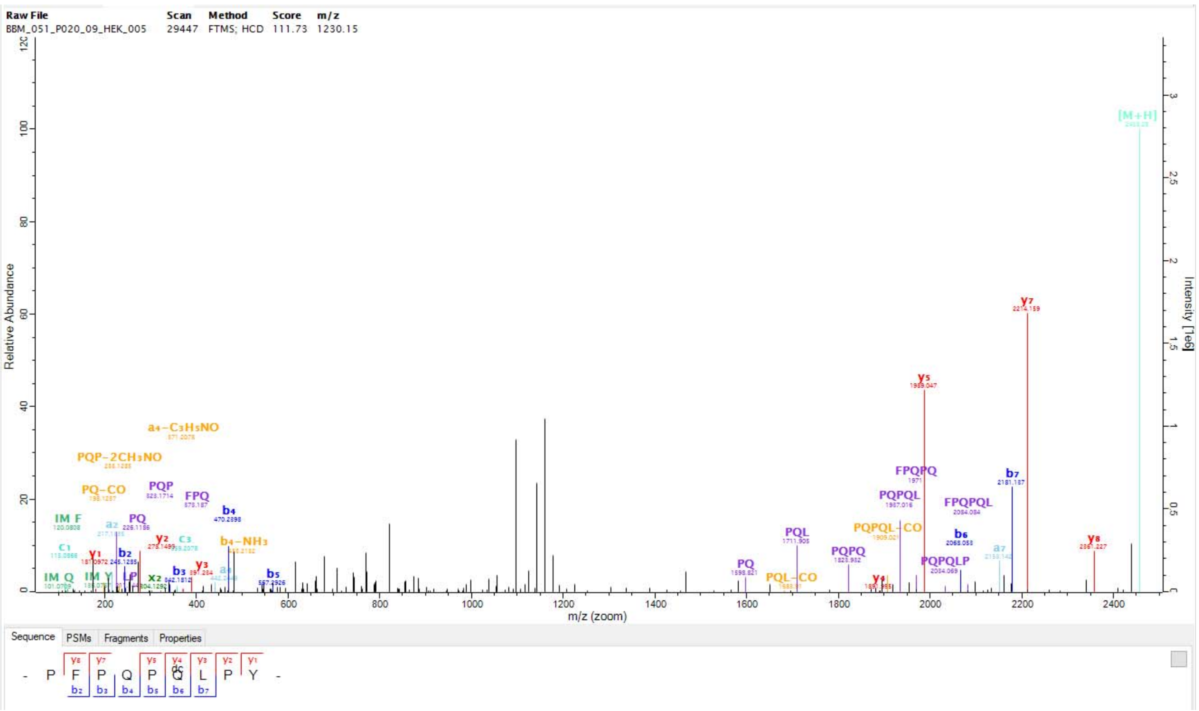
Raw File Scan Method Score m/z
BBM_051_P020_09_HEK_005 29447 FTMS; HCD 111.73 1230.15
Relative Abundance
Intensity [1e6]
m/z (zoom)
Sequence PSMs Fragments Properties
- P F P Q P Q L P Y -

U

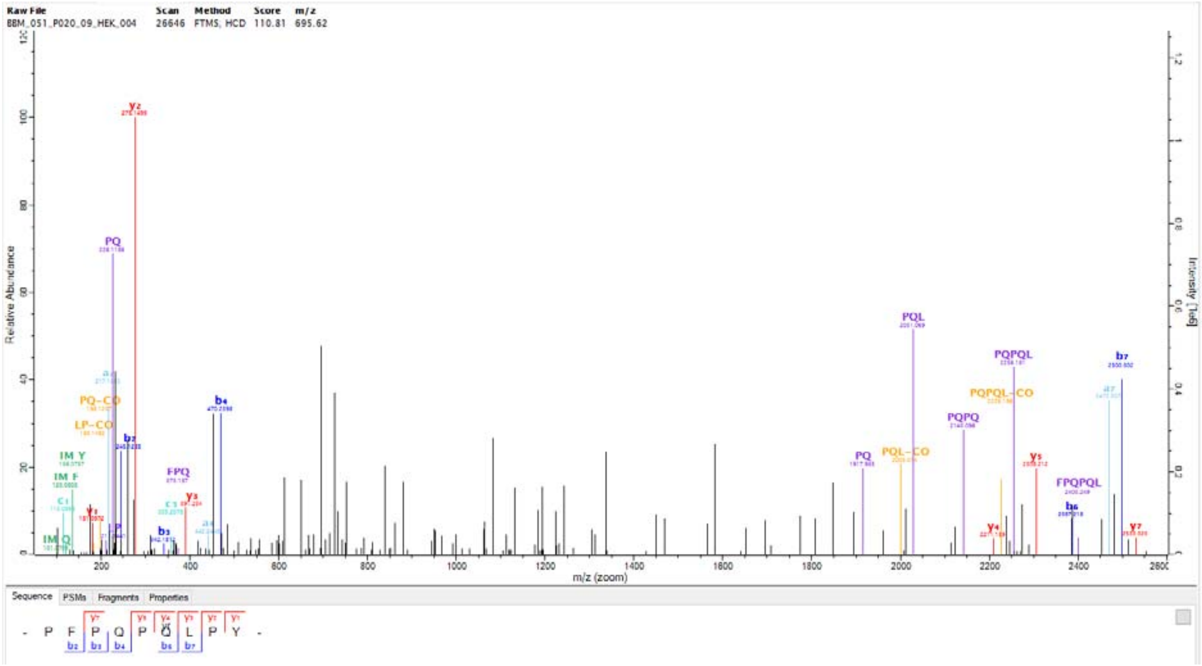
Raw File Scan Method Score m/z
BBM_051_P020_09_HEK_004 26646 FTMS; HCD 110.81 695.62
Relative Abundance
Intensity [1e6]
m/z (zoom)
Sequence PSMs Fragments Properties
- P F P Q P Q L P Y -

V

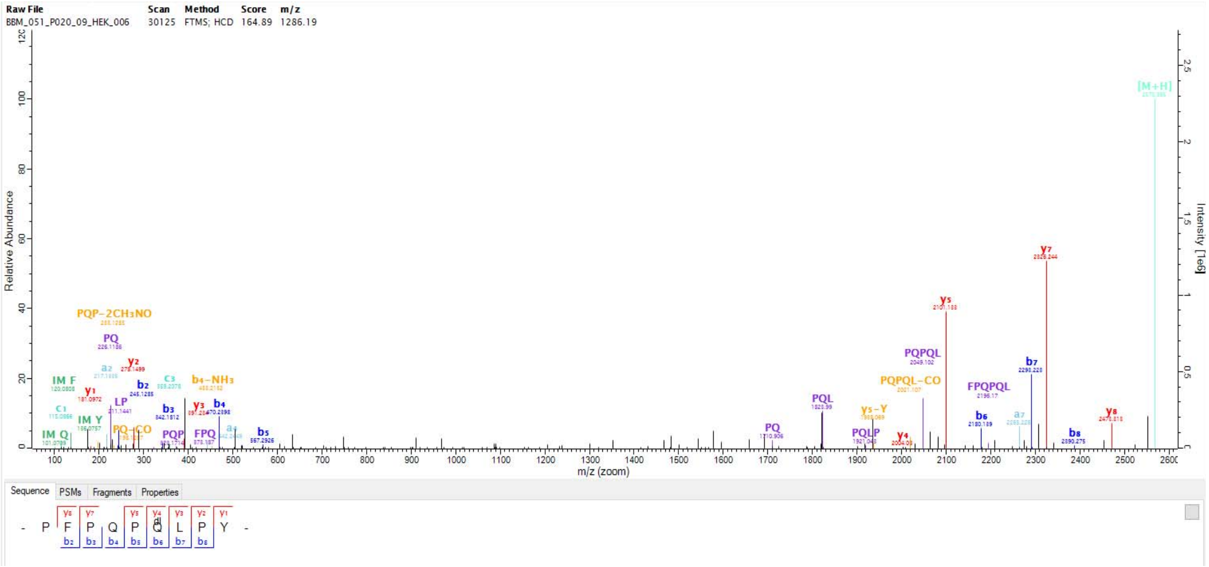
Raw File Scan Method Score m/z
BBM_051_P020_09_HEK_006 30125 FTMS; HCD 164.89 1286.19
Relative Abundance
Intensity [1e6]
m/z (zoom)
Sequence PSMs Fragments Properties
- P F P Q P Q L P Y -

W

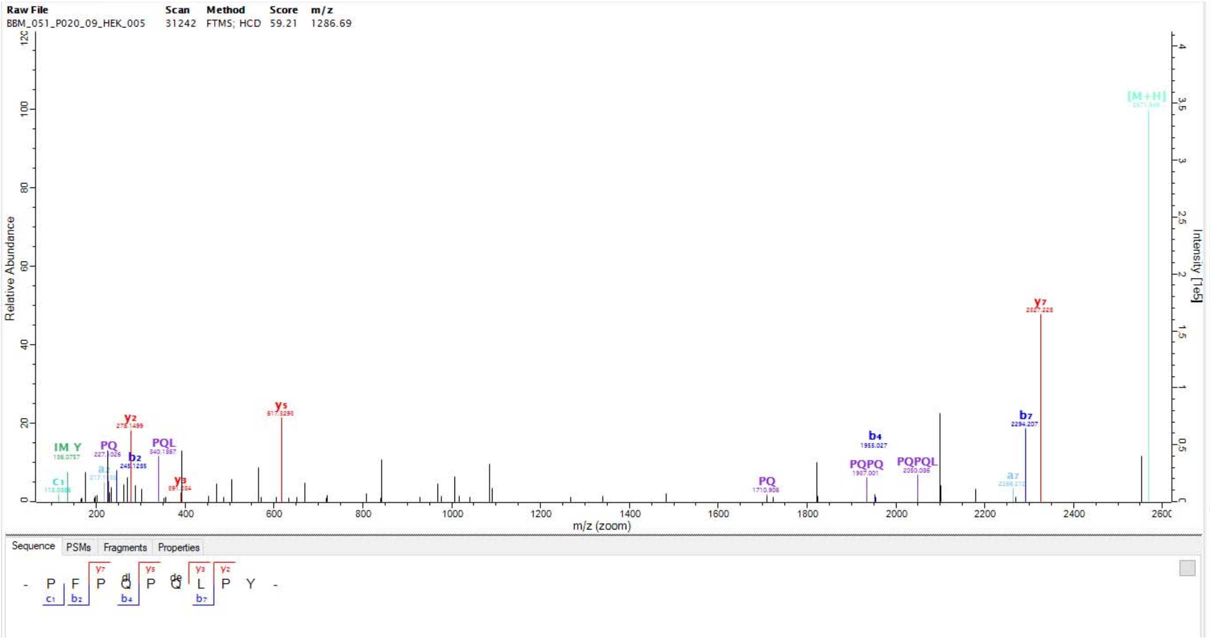
Raw File Scan Method Score m/z
BBM_051_P020_09_HEK_005 31242 FTMS; HCD 59.21 1286.69
Relative Abundance
Intensity [1e5]
m/z (zoom)
[M+H]
y7
b7
b4
PQPQ
PQPQL
PQ
y5
y2
b2
y3
PQL
IM Y
c1
a7
Sequence PSMs Fragments Properties

X

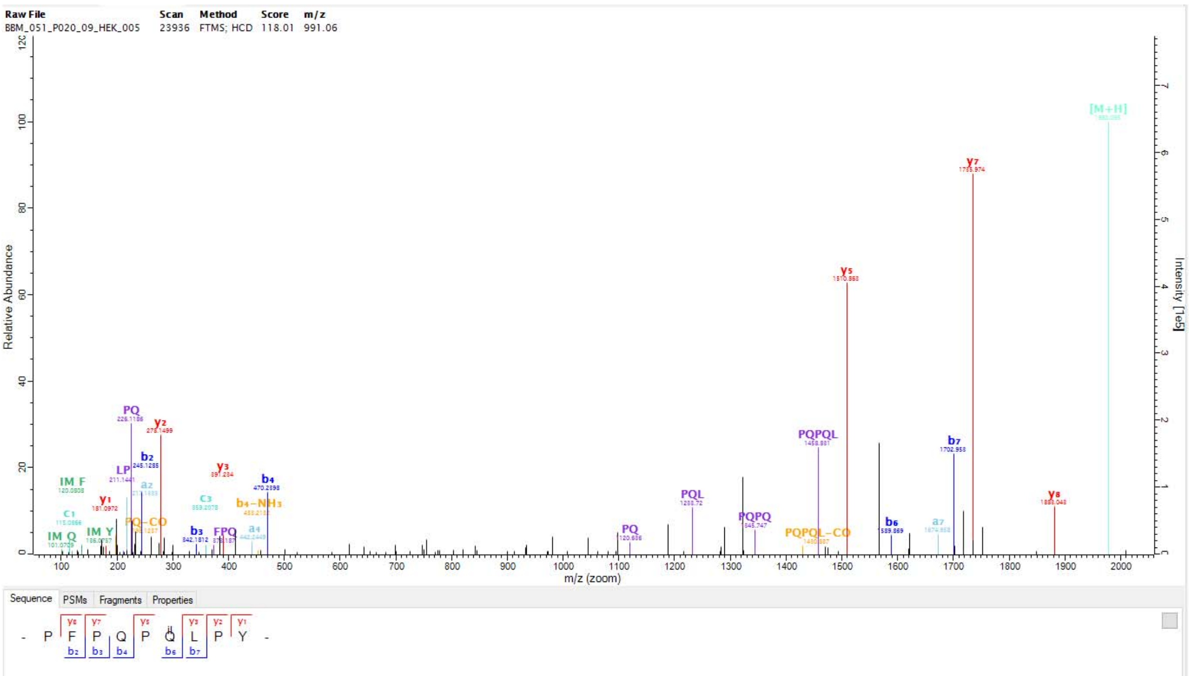
Raw File Scan Method Score m/z
BBM_051_P020_09_HEK_005 23936 FTMS; HCD 118.01 991.06
Relative Abundance
Intensity [1e5]
m/z (zoom)
[M+H]
y7
y5
PQPQL
b7
y8
PQL
PQPQ
PQPQL-CO
b6
a7
PQ
y2
y3
b4
b4-NH3
b2
LP
a2
IM F
y1
c1
c3
PQ-CO
b3
FPQ
a4
IM Q
IM Y
Sequence PSMs Fragments Properties

Y

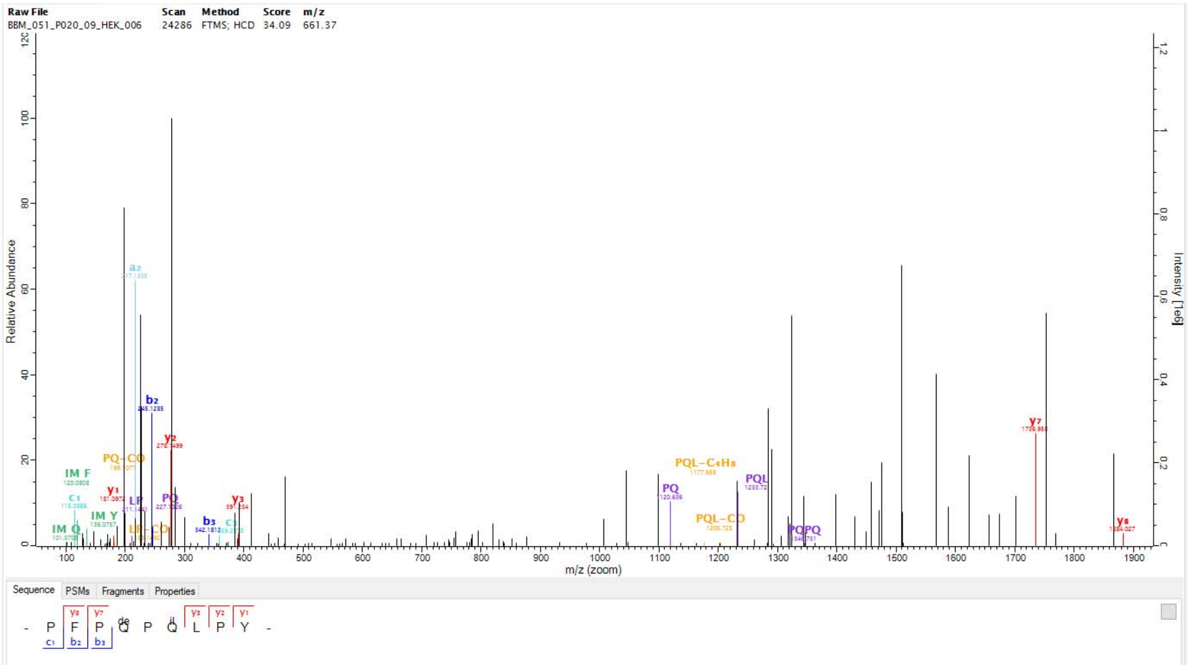
Raw File Scan Method Score m/z
BBM_051_P020_09_HEK_006 24286 FTMS; HCD 34.09 661.37
Relative Abundance
Intensity [1e6]
m/z (zoom)
Sequence PSMs Fragments Properties
- P F P Q P Q L P Y -

Z

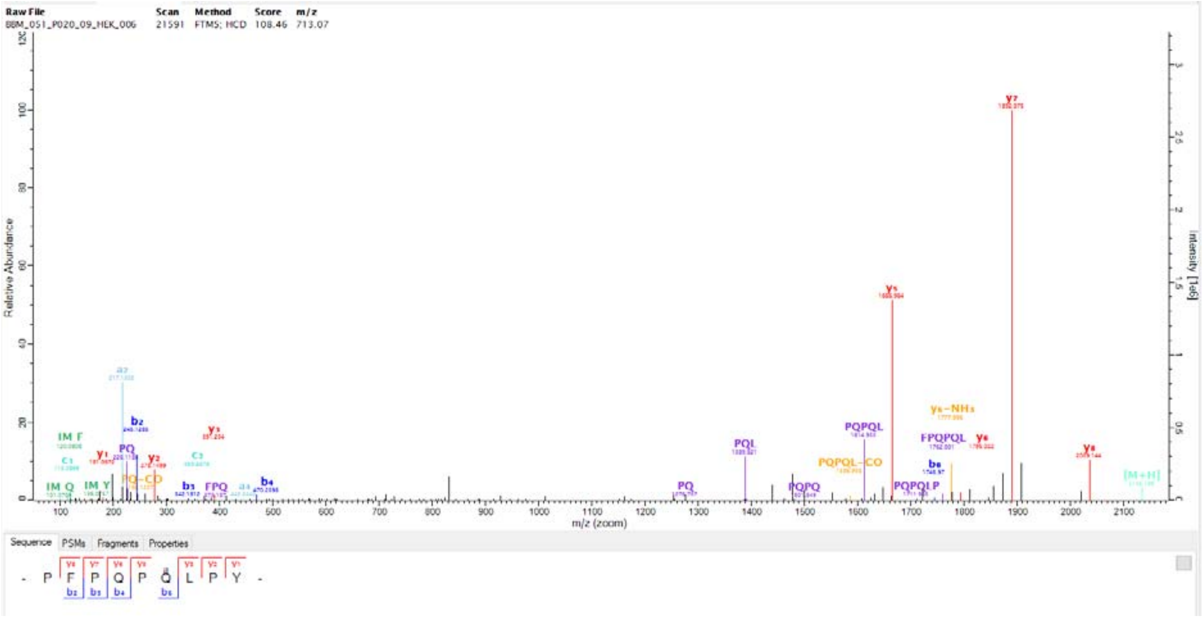
Raw File Scan Method Score m/z
BBM_051_P020_09_HEK_006 21591 FTMS; HCD 108.46 713.07
Relative Abundance
Intensity [1e6]
m/z (zoom)
Sequence PSMs Fragments Properties
- P F P Q P Q L P Y -

AA

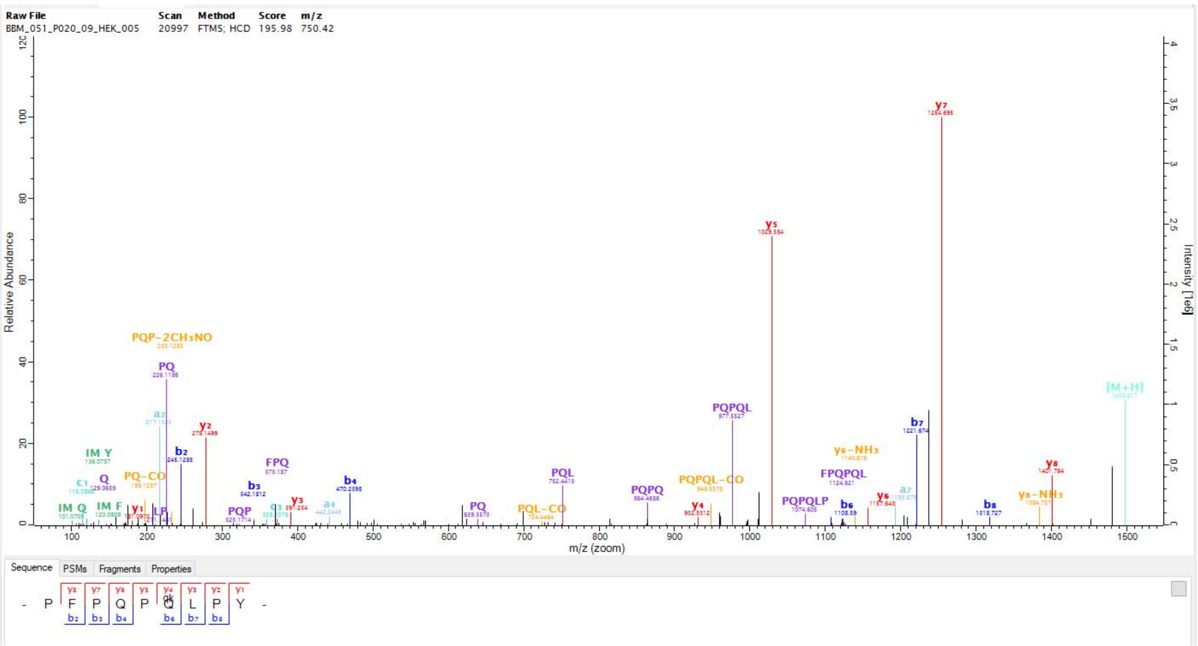
Raw File
BBM_051_P020_09_HEK_005
Scan
20997
Method
FTMS; HCD
Score
195.98
m/z
750.42
Relative Abundance
Intensity [1e6]
m/z (zoom)
y7
y5
PQP-2CH3NO
PQ
a2
y2
b2
IM Y
Q
PQ-CO
c1
IM Q
IM F
y1
LP
FPQ
b3
PQP
c3
y3
a4
b4
PQ
PQL-CO
PQL
PQPQ
PQPQL-CO
y4
PQPQL
y6-NH3
FPQPQL
PQPQLP
b6
y6
a7
b7
b8
y8-NH3
y8
[M+H]
Sequence
PSMs
Fragments
Properties
- P F P Q P Q L P Y -

AB

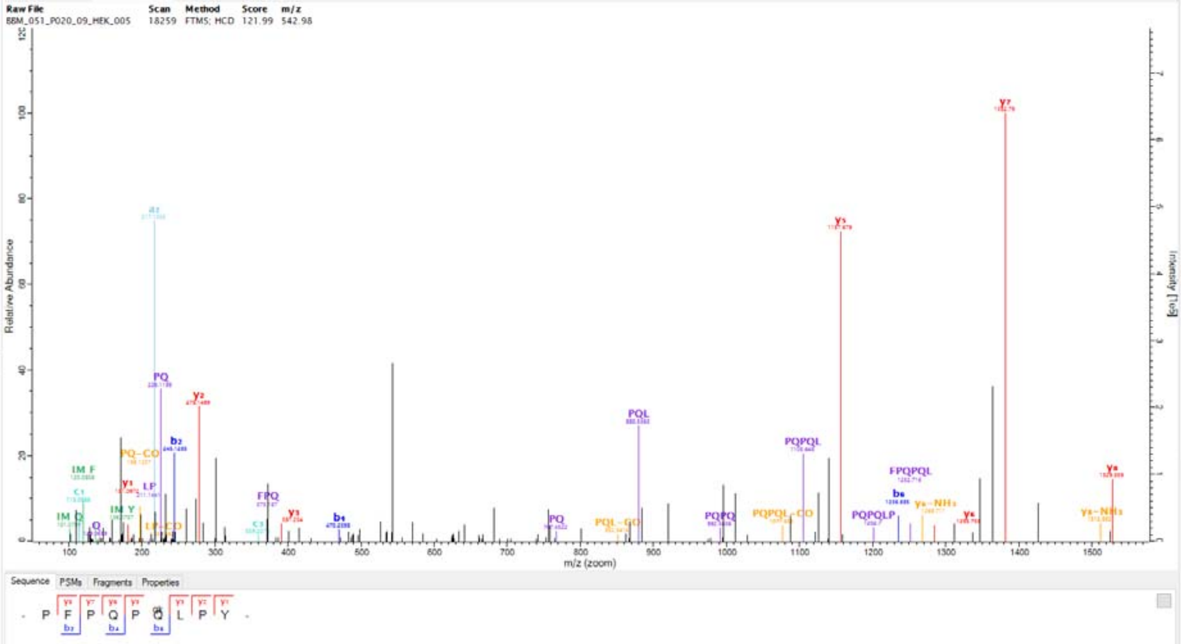
Raw File
BBM_051_P020_09_HEK_005
Scan
18259
Method
FTMS; HCD
Score
121.99
m/z
542.98
Relative Abundance
Intensity [1e6]
m/z (zoom)
y7
y5
a2
PQ
y2
PQL
b2
PQ-CO
IM F
c1
y1
LP
IM Q
IM Y
FPQ
y3
c3
b4
PQ
PQL-CO
PQPQ
PQPQL-CO
PQPQL
PQPQLP
FPQPQL
b6
y6-NH3
y6
y8
y8-NH3
Sequence
PSMs
Fragments
Properties
- P F P Q P Q L P Y -

AC

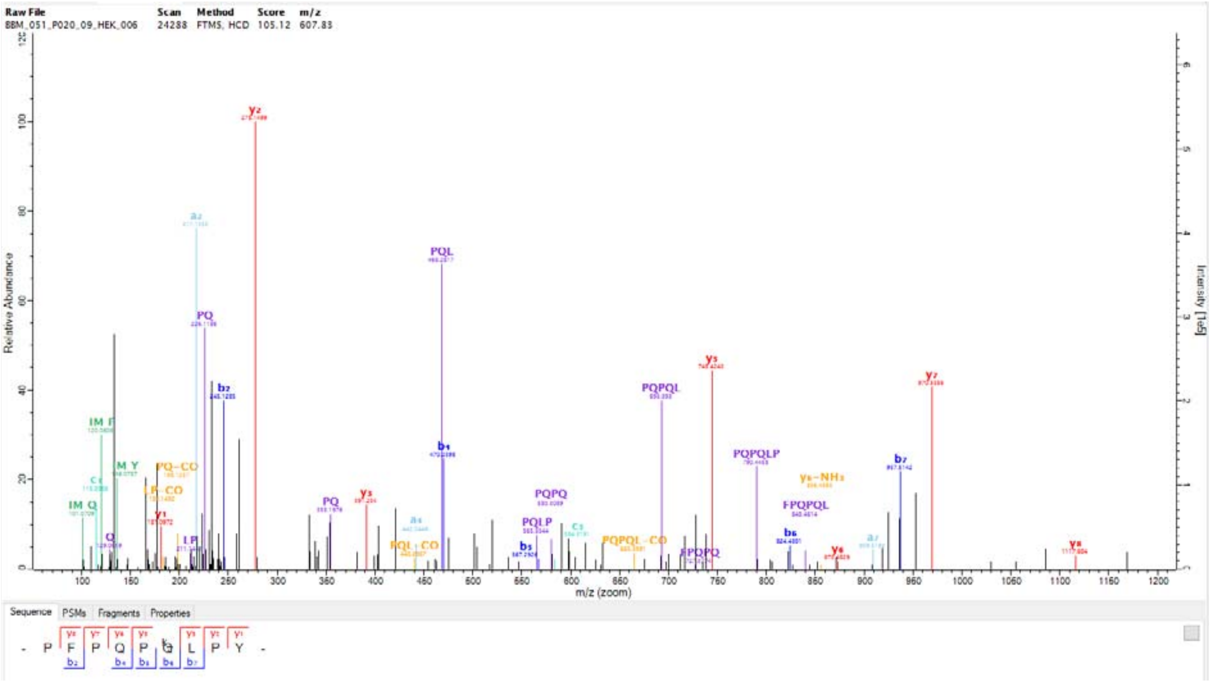
Raw File Scan Method Score m/z
BBM_051_P020_09_HEK_006 24288 FTMS; HCD 105.12 607.83
Relative Abundance
Intensity [1e5]
m/z (zoom)

AD

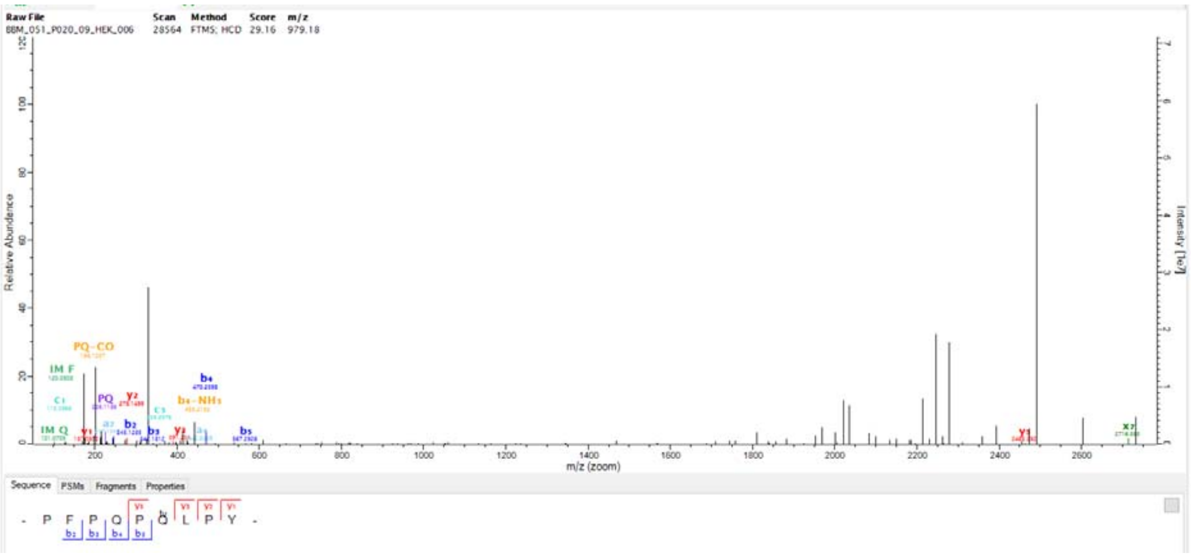
Raw File Scan Method Score m/z
BBM_051_P020_09_HEK_006 28564 FTMS; HCD 29.16 979.18
Relative Abundance
Intensity [1e7]
m/z (zoom)

AE

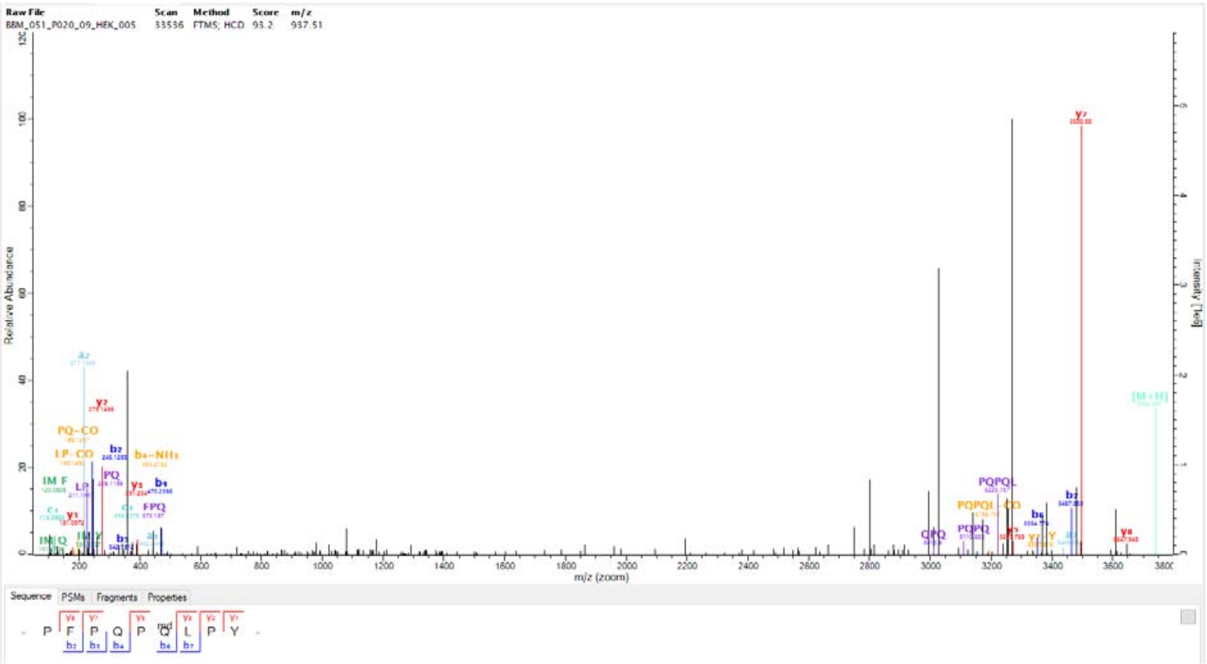
Raw File
BBM_051_P020_09_HEK_005
Scan
33536
Method
FTMS; HCD
Score
93.2
m/z
937.51
Relative Abundance
Intensity [1e6]
m/z (zoom)
Sequence
PSMs
Fragments
Properties

AF

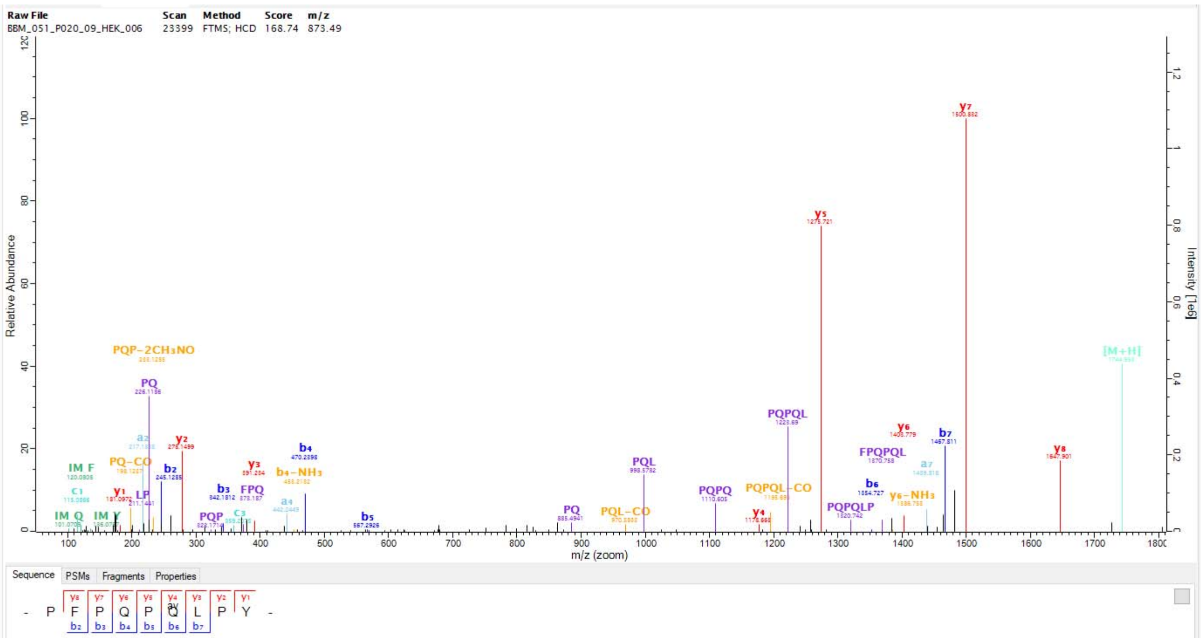
Raw File
BBM_051_P020_09_HEK_006
Scan
23399
Method
FTMS; HCD
Score
168.74
m/z
873.49
Relative Abundance
Intensity [1e6]
m/z (zoom)
Sequence
PSMs
Fragments
Properties

AG

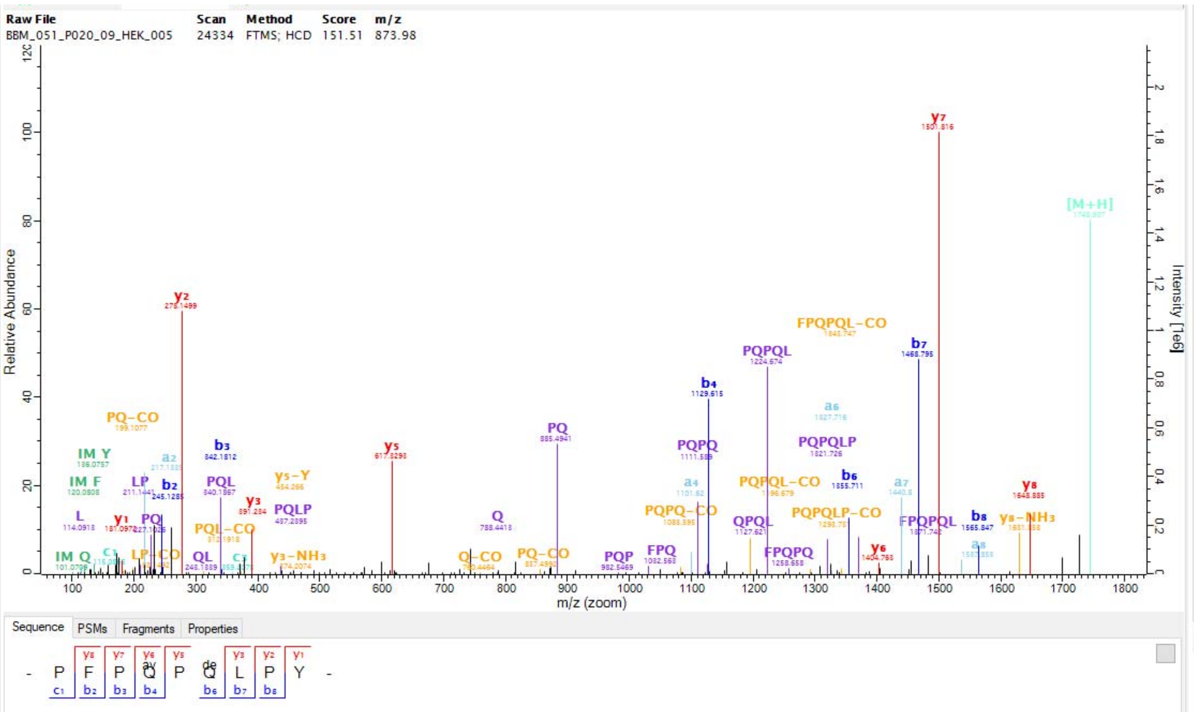

Raw File Scan Method Score m/z
BBM_051_P020_09_HEK_005 24334 FTMS; HCD 151.51 873.98
Relative Abundance
Intensity [1e6]
m/z (zoom)
Sequence PSMs Fragments Properties


AH

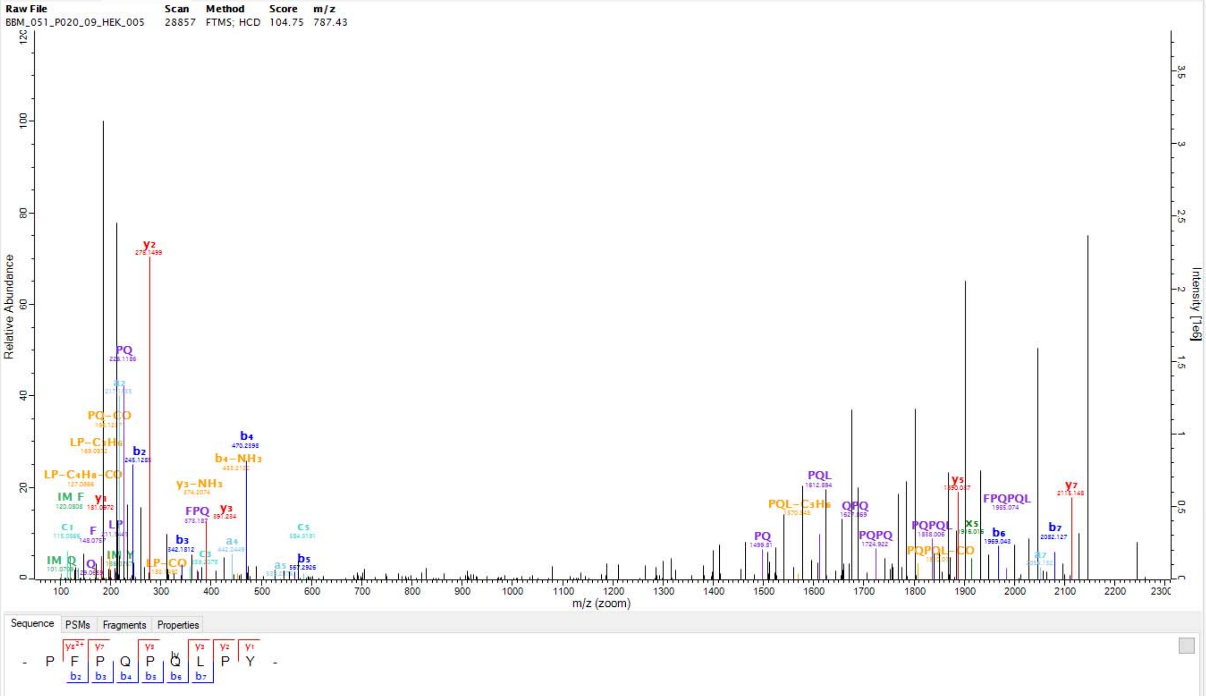

Raw File Scan Method Score m/z
BBM_051_P020_09_HEK_005 28857 FTMS; HCD 104.75 787.43
Relative Abundance
Intensity [1e6]
m/z (zoom)
Sequence PSMs Fragments Properties

AI

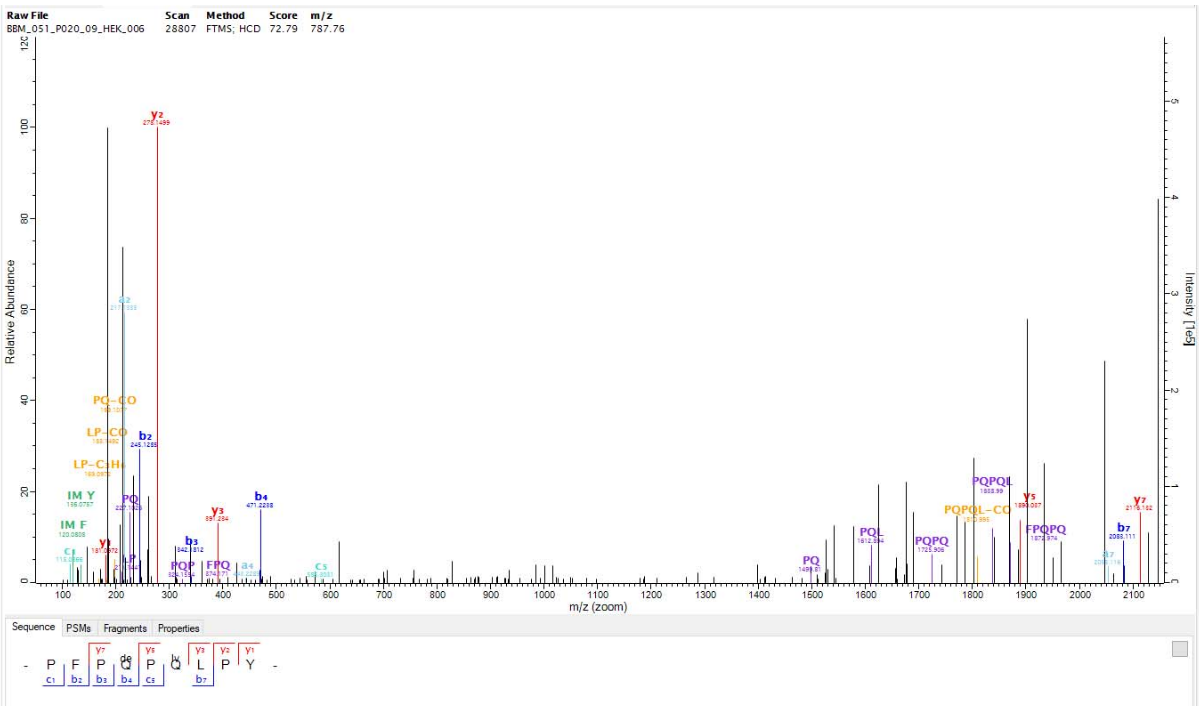
Raw File Scan Method Score m/z
BBM_051_P020_09_HEK_006 28807 FTMS; HCD 72.79 787.76
Relative Abundance
Intensity [1e5]
m/z (zoom)
Sequence PSMs Fragments Properties

AJ

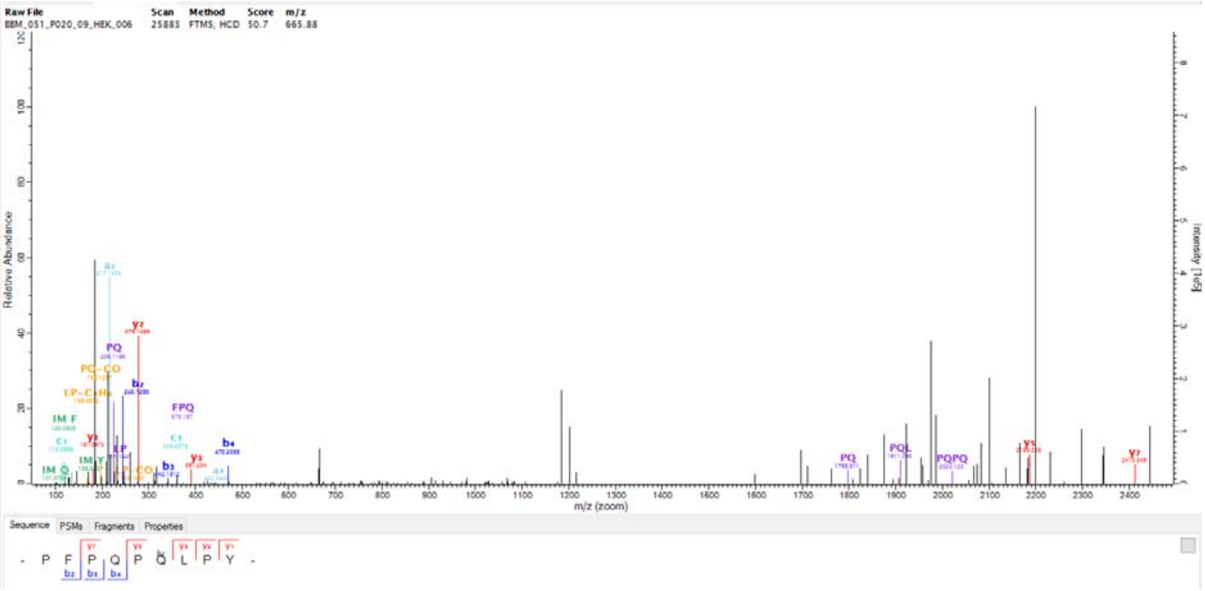
Raw File Scan Method Score m/z
BBM_051_P020_09_HEK_006 25883 FTMS; HCD 50.7 665.88
Relative Abundance
Intensity [1e5]
m/z (zoom)
Sequence PSMs Fragments Properties

Supplemental Figure S3. **MS/MS spectra of isopeptides between human tissue transglutaminase (TG2) and PepQ (PFPQPQLPY) or its deamidated form PepE.** The fragments are marked in different colors as follows: y-fragments in red; b-fragments in blue; a- and c-fragments in turquoise; internal fragments in violet or green (single amino acids); fragments with losses of NH3 or CO marked in orange. For every identified isopeptide a single MS/MS spectrum with annotated fragments is shown:

*A*, QEYVLTQQGFIYQGSAKFIK/PepE
*B*, FLKNAGR/PepQ
*C*, FLKNAGR/PepE
*D*, WKNHGCQR/PepQ
*E*, AIKEGDLSTK/PepQ
*F*, AIKEGDLSTK/PepE
*G*, SLIVGLKISTK/PepQ
*H*, ISTKSVGR/PepQ
*I*, ISTKSVGRDER/PepQ
*J*, EDITHTYKYPEGSSEER/PepQ
*K*, EDITHTYKYPEGSSEER/PepE
*L*, DEREDITHTYKYPEGSSEER/PepQ
*M*, LEAKEETGMAMR/PepQ
*N*, LEAKEETGMAMR/PepE
*O*, ANHLNKLEAKEETGMAMR/PepQ
*P*, ANHLNKLEAKEETGMAMR/Pep
*Q*, ANHLNK/PepQ
*R*, ANHLNK/PepE
*S*, SVPLCILYEKYR/PepQ
*T*, DCLTESNLIKVR/PepQ
*U*, YRDCLTESNLIKVR/PepQ
*V*, DLYLENPEIKIR/PepQ
*W*, DLYLENPEIKIR/PepE
*X*, ILGEPKQK/PepQ
*Y*, ILGEPKQK/PepE
*Z*, ILGEPKQKR/PepQ
*AA*, QKR/PepQ
*AB*, QKRK/PepQ
*AC*, K/PepQ
*AD*, TVEIPDPVEAGEEVKVR/PepQ
*AE*, MDLLPLHMGLHKLVVNFESDKLK/PepQ
*AF*, AVKGFR/PepQ
*AG*, AVKGFR/PepE
*AH*, LVVNFESDKLK/PepQ
*AI*, LVVNFESDKLK/PepE
*AJ*, LVVNFESDKLKAVK/PepQ.
