# Supplementary material for: Comprehensive Detection of Isopeptides between Human Tissue Transglutaminase and Gluten Peptides
Source: Nutrients. 2019 Sep 20;11(10):2263. doi: 10.3390/nu11102263 (PMC6835481; doi:10.3390/nu11102263)
Supplement: Supplementary file 1 [file nutrients-11-02263-s001.zip › supplM/isopeptides_tab_s1.pdf]

Supplementary Table S1

TG2 peptides containing lysine residues identified as isopeptide crosslinking sites

Lysine No.	Sequence of TG2 peptide	Formula of the modification in MaxQuant and Skyline[a]	Modification mass
173	QEYVLTQQGFIYQGSA**K**FI	$C_{110}H_{163}N_{25}O_{31}$	2330.19
205	FL**K**NAGR	$C_{36}H_{57}N_{11}O_{9}$	787.43
265	W**K**NHGCQR	$C_{43}H_{62}N_{16}O_{11}S$	1010.45
380	AI**K**EGDLSTK	$C_{45}H_{77}N_{11}O_{17}$	1043.55
425	SLIVGL**K**ISTK	$C_{53}H_{96}N_{12}O_{15}$	1140.71
429	IST**K**SVGR	$C_{35}H_{63}N_{11}O_{12}$	829.47
429	IST**K**SVGRDER	$C_{50}H_{87}N_{17}O_{19}$	1229.64
444	EDITHTY**K**YPEGSSEER	$C_{87}H_{126}N_{22}O_{34}$	2022.88
444	DEREDITHTY**K**YPEGSSEE	$C_{102}H_{150}N_{28}O_{41}$	2423.05
464	ANHLN**K**LAEKEETGMAM	$C_{84}H_{140}N_{26}O_{28}S_{2}$	2024.98
464	ANHLN**K**LAEK	$C_{49}H_{81}N_{15}O_{15}$	1119.60
468	LAE**K**EETGMAMR	$C_{55}H_{93}N_{15}O_{20}S_{2}$	1347.62
550	SVPLCILYE**K**YR	$C_{68}H_{107}N_{15}O_{18}S$	1453.76
562	DCLTESNLI**K**VR	$C_{58}H_{100}N_{16}O_{20}S$	1372.70
562	YRDCLTESNLI**K**VR	$C_{73}H_{121}N_{21}O_{23}S$	1691.87
590	DLYLENPEI**K**IR	$C_{68}H_{108}N_{16}O_{21}$	1484.79
598	ILGEP**K**QK	$C_{41}H_{70}N_{10}O_{12}$	894.52
598	ILGEP**K**QKR	$C_{47}H_{82}N_{14}O_{13}$	1050.62
600	Q**K**R	$C_{17}H_{31}N_{7}O_{5}$	413.24
600	Q**K**RK	$C_{23}H_{43}N_{9}O_{6}$	541.33
602	**K**	$C_{6}H_{11}N_{1}O_{2}$	129.08
649	TVEIPDPVEAGEEV**K**VR	$C_{81}H_{132}N_{20}O_{29}$	1848.94
663	MDLLPLHMGLH**K**LVVNF	$C_{122}H_{198}N_{30}O_{32}S_{2}$	2659.42
672	LVVNFESD**K**LK	$C_{59}H_{95}N_{13}O_{18}$	1273.69
672/ 674	LVVNFESDKL**K**AVK	$C_{73}H_{121}N_{17}O_{21}$	1571.89
677	AV**K**GFR	$C_{31}H_{49}N_{9}O_{7}$	659.38
**Glutamine No.**	**Sequence of PepQ**	**Formula of the modification in MaxQuant and Skyline[a]**	**Modification mass**
6	PFPQPQLPY-NH_2	$C_{54}H_{73}N_{11}O_{12}$	1067.54

[a] Formula of TG2 peptide with NH_3 subtracted to account for the isopeptide bond
