# Supplementary material for: Comprehensive Detection of Isopeptides between Human Tissue Transglutaminase and Gluten Peptides
Source: Nutrients. 2019 Sep 20;11(10):2263. doi: 10.3390/nu11102263 (PMC6835481; doi:10.3390/nu11102263)
Supplement: Supplementary file 1 [file nutrients-11-02263-s001.zip › supplM/isopeptides_tab_s2.pdf]

Supplementary Table S2

Alignment of the crosslinking sites of TG2 sorted by most preferred to least preferred in the model system. The crosslinked lysine residues as well as the four surrounding amino acids are indicated.

**Lysine residue**[a]					**K**				
**A Most preferred lysine residues**									
**425**	I	V	G	L	**K**	I	S	T	K
**590**	N	P	E	I	**K**	I	R	I	L
**600**	E	P	K	Q	**K**	R	K	L	V
**649**	G	E	E	V	**K**	V	R	M	D
**B Less preferred lysine residues**									
**205**	K	P	F	L	**K**	N	A	G	R
**464**	N	H	L	N	**K**	L	A	E	K
**562**	S	N	L	I	**K**	V	R	A	L
**598**	L	G	E	P	**K**	Q	K	R	K
**672**	F	E	S	D	**K**	L	K	A	V
**677**	L	K	A	V	**K**	G	F	R	N
**C Least preferred lysine residues**									
**173**	Q	G	S	A	**K**	F	I	K	N
**265**	L	R	R	W	**K**	N	H	G	C
**380**	V	R	A	I	**K**	E	G	D	L
**429**	K	I	S	T	**K**	S	V	G	R
**444**	T	H	T	Y	**K**	Y	P	E	G
**468**	K	L	A	E	**K**	E	E	T	G
**550**	I	L	Y	E	**K**	Y	R	D	C
**602**	K	Q	K	R	**K**	L	V	A	E
**663**	M	G	L	H	**K**	L	V	V	N
**674**	S	D	K	L	**K**	A	V	K	G

[a] Position of the lysine residue in the amino acid sequence of human tissue transglutaminase (TG2, P21980)

Amino acids in black belong to the peptide sequence identified as part of the isopeptide, those in grey are the ones following in the amino acid sequence of TG2 before or after the tryptic cleavage site.
